# Reduced expression of Paternally Expressed Gene-3 enhances somatic cell reprogramming through mitochondrial activity perturbation

**DOI:** 10.1038/s41598-017-10016-7

**Published:** 2017-08-29

**Authors:** Ilda Theka, Francesco Sottile, Francesco Aulicino, Alvaro Castells Garcia, Maria Pia Cosma

**Affiliations:** 1 Centre for Genomic Regulation (CRG), The Barcelona Institute of Science and Technology, Dr. Aiguader 88, 08003 Barcelona, Spain; 2 Universitat Pompeu Fabra (UPF), 08003 Barcelona, Spain; 3 Institució Catalana de Recerca i Estudis Avançats (ICREA), Pg. Lluís Companys 23, 08010 Barcelona, Spain

## Abstract

Imprinted genes control several cellular and metabolic processes in embryonic and adult tissues. In particular, paternally expressed gene-3 (Peg3) is active in the adult stem cell population and during muscle and neuronal lineage development. Here we have investigated the role of Peg3 in mouse embryonic stem cells (ESCs) and during the process of somatic cell reprogramming towards pluripotency. Our data show that Peg3 knockdown increases expression of pluripotency genes in ESCs and enhances reprogramming efficiency of both mouse embryonic fibroblasts and neural stem cells. Interestingly, we observed that altered activity of Peg3 correlates with major perturbations of mitochondrial gene expression and mitochondrial function, which drive metabolic changes during somatic cell reprogramming. Overall, our study shows that Peg3 is a regulator of pluripotent stem cells and somatic cell reprogramming.

## Introduction

Paternally expressed gene-3 (*Peg3*, also known as *Pw1*) is an imprinted gene that encodes a Krüppel-type (C2H2) zinc finger protein^1^. During embryo development, *Peg3* expression is detected upon gastrulation, where it plays a major role in muscle and neuronal lineage development^1, 2^. In adult tissues, *Peg3* is expressed in several stem cell compartments, thus it has been considered a marker for competent self-renewing adult stem cells^3^. Moreover defects in *PEG3* expression have been identified in human gynecologic cancer^4, 5^ and glioma cell lines^6^. Accordingly, *PEG3* plays a tumor suppressor role in many types of cancers and exogenous *PEG3* overexpression in glioma cell lines resulted in loss of tumorigenicity^7^. Furthermore, *Peg3* is involved in apoptosis induction, more specifically it can contribute to p53 activation and Bax translocation^8, 9^. Nevertheless, the mechanisms behind *Peg3* action in different cellular contexts (both in embryonic and adult tissues) still remain obscure. Peg3 could possibly control cell proliferation^10^ and cellular metabolism by directly regulating mitochondrial gene expression^11^. Indeed, Peg3 can bind and control the expression of mitochondrial related genes, including *Tufm*, *Slc25a29*, *Mrpl45*, among others^12^. Mitochondria not only provide energy to the cells but also control several molecular processes, such as cell growth, cell cycle, and cell death^13^. Moreover mitochondrial activity determines and regulates metabolic states that are important for the maintenance of cell homeostasis and for driving cell fate transition^14^.

Here we investigated the role of Peg3 in pluripotent mouse embryonic stem cells (ESCs), which are highly proliferative and characterized by a glycolytic metabolism. During ESC differentiation a transition towards oxidative phosphorylation (OXPHOS) occurs and thus correlates with high mitochondria activity^15–19^. In contrast, the metabolic switch from OXPHOS towards glycolysis has been shown to occur during somatic cell reprogramming^18–20^. We show that low levels of Peg3 are associated with pluripotency and enhance somatic cell reprogramming efficiency by modulating mitochondrial activity.

## Results

### Peg3/Pw1 endogenous expression levels anti-correlate with pluripotency state

**Figure 1:**
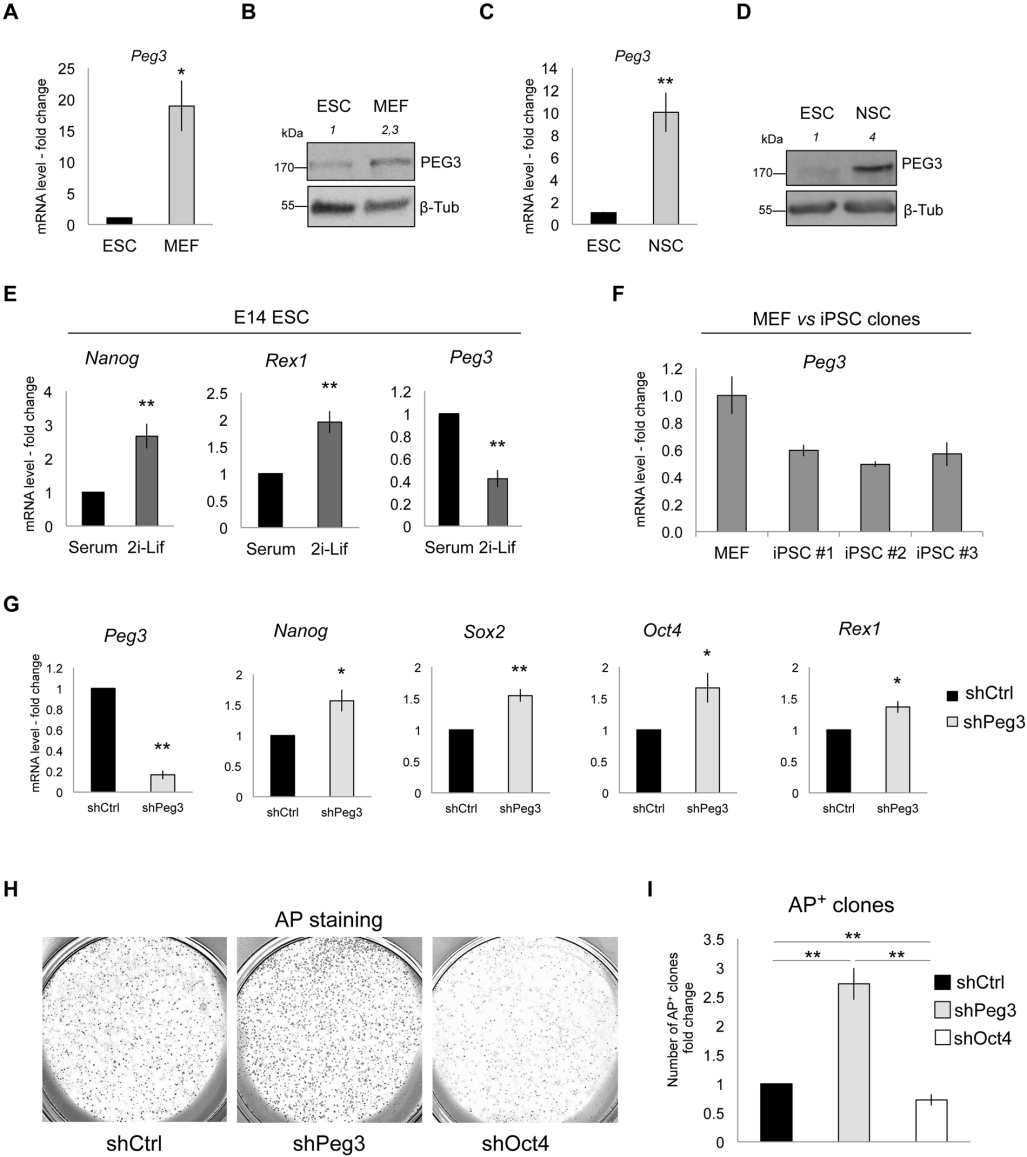
Peg3 expression levels anti-correlate with pluripotency state. (**A,C**) Quantitative real-time PCR analysis of endogenous *Peg3* expression levels in ESCs, MEFs (**A**) and NSCs (**C**) (n = 3 independent experiments). (**B,D**) Representative western-blots (out of n = 2 independent experiments) showing endogenous PEG3 protein levels in ESCs *versus* MEFs (**B**) and *versus* NSCs (**D**). (**E,F**) Quantitative real-time PCR analysis showing endogenous *Peg3* expression in 2i-Lif media (2i-Lif) *versus* serum-Lif (Serum) (n = 3 independent experiments) (**E**), and in induced pluripotent stem cell (iPSC) clones *versus* MEFs (n = 3 technical replicates) (**F**). (**G)** Quantitative real-time PCR analysis of pluripotency gene expression levels in ESCs after *Peg3* stable silencing (n = 3 independent experiments). (**H,I**) Representative bright field and quantification of alkaline phosphatase positive (AP^+^) colonies represented as fold change and counted at day 7 of the AP^+^-CFC assay (n = 3 independent experiments). shOct4 silencing construct was used as a negative control. (**A**,**C**,**E**,**G**,**I**) Data are represented as fold change (2^−ΔΔCt^) and means of n = 3 independent experiments ± SE. Asterisks indicate statistical significance calculated by unpaired two-tailed t test analysis (*p < 0.05; **p < 0.01). For western-blot analysis β-Tubulin was used as loading control and densitometric analysis was carried out by using ImageJ software. The quantification reflects the relative amounts as a ratio of each protein band relative to their loading control.

Peg3/Pw1 is expressed in several adult tissues and in particular in adult stem cells^3^. However, its involvement in the regulation of molecular processes controlling pluripotency has not been described. To investigate this possibility, we first analyzed its endogenous expression levels in E14 mouse embryonic stem cells (ESCs). Peg3 was expressed at very low levels in ESCs when compared to both mouse embryonic fibroblasts (MEFs) and neural stem cells (NSCs), both at mRNA and protein level (Fig. 1A–D). In addition, both *Peg3* transcript and protein levels strongly increased during embryoid bodie (EB) formation (Supplementary Figure 1A and B) and in E14 ESCs differentiated toward NSCs^21, 22^ (Supplementary Figure 1C and D).

Interestingly, even though *Peg3* was expressed at very low levels in ESCs, we observed a further transcriptional downregulation when ESCs were grown in 2i-Lif medium (2i-Lif) for 4 days as compared to the serum-Lif medium (Serum) (Fig. 1E). ESCs grown in 2i-Lif medium (a defined serum free ESC medium containing the GSK3β and MEK inhibitors)^23^, can reach the ground state of pluripotency, which resembles the inner cell mass (ICM)^24^, as confirmed by the increased expression of pluripotency genes such as *Nanog* and *Rex1* (Fig. 1E). Moreover, *Peg3* transcriptional levels decreased in induced pluripotent stem cell (iPSCs) when compared to MEFs (Fig. 1F). These observations indicate that Peg3 levels anti-correlate with pluripotency state and are higher in differentiated cells.

### Peg3 silencing positively regulates pluripotency and self-renewal of mouse ESCs

Given that *Peg3* increased at the onset of differentiation and it was downregulated in ESCs cultured in 2i-Lif medium, we asked whether *Peg3* knockdown (KD) could affect self-renewal and pluripotency. To address this hypothesis we silenced Peg3 (shPeg3) in ESCs, both stably and transiently, by using either pLKO-shCtrl (a vector carrying a control short hairpin with no predicted targets in the mouse transcriptome) or pLKO-shPeg3 construct, which carries a short hairpin targeting Peg3. Both shCtrl and shPeg3 contructs contain a hygromycin resistance cassette, which allow us to select the infected cells^25^. The pluripotency markers *Nanog*, *Sox2*, *Oct4* and *Rex1* increased after *Peg3* downregulation (Fig. 1G and Supplementary Figure 1E). In addition, to analyze the effect of Peg3 KD on ESC self-renewal, we performed alkaline phosphatase colony-forming cell (AP-CFC) assay. The alkaline phosphatase membrane-bound enzyme is one of the key markers to identify pluripotent stem cells, such as ESCs^26^. Interestingly *Peg3* transient inhibition resulted in higher number of alkaline phosphatase positive (AP^+^) ESC colonies when compared to the control (shCtrl). On the contrary, the number of AP^+^ colonies decreased after Oct4 transient silencing, which we used as negative control (Fig. 1H and I). Oct4 has been described to impair ESC self-renewal capability^27^, which is consistent with our data. Moreover, *Peg3* transient KD induced the expression of the pluripotency markers, such as *Nanog* and *Oct4*, which, on the contrary, were downregulated after Oct4 depletion (Supplementary Figure 1E).

To further confirm its effect on pluripotency, we stably silenced *Peg3* in another ESC line (REX1-dGFP ESCs), which carried a destabilized GFP (dGFP) under the control of the endogenous Rex1 promoter^23^. REX1-dGFP ESCs carrying *Peg3* KD displayed significant increase in the percentage of GFP positive (GFP^+^) cells when compared to control (shCtrl), accordingly the number of GFP negative (GFP^−^) cells decreased (Supplementary Figure 1F). However, even though, *Peg3* KD resulted in higher percentage of GFP^+^ cells, its depletion alone was not sufficient to induce the ground pluripotency state in ESCs. Indeed, the GFP^+^ cells in 2i-Lif medium were around 95 percent, as previously reported^23^, in both shCtrl and shPeg3 infected ESCs (Supplementary Figure 1F). Moreover, when we induced ESC differentiation in NSCs the difference in the GFP^+^/GFP^−^ ratio between shCtrl and shPeg3 conditions was maintained over time, suggesting a delay in the differentiation potential of the Peg3 depleted ESCs (Supplementary Figure 1G). Of note, as in the case of E14 (Fig. 1G), *Peg3* KD was efficient in REX1-dGFP ESCs and it was maintained silenced during differentiation (Supplementary Figure 1H and I).

### Peg3 silencing enhances somatic cell reprogramming of MEFs and NSCs

**Figure 2:**
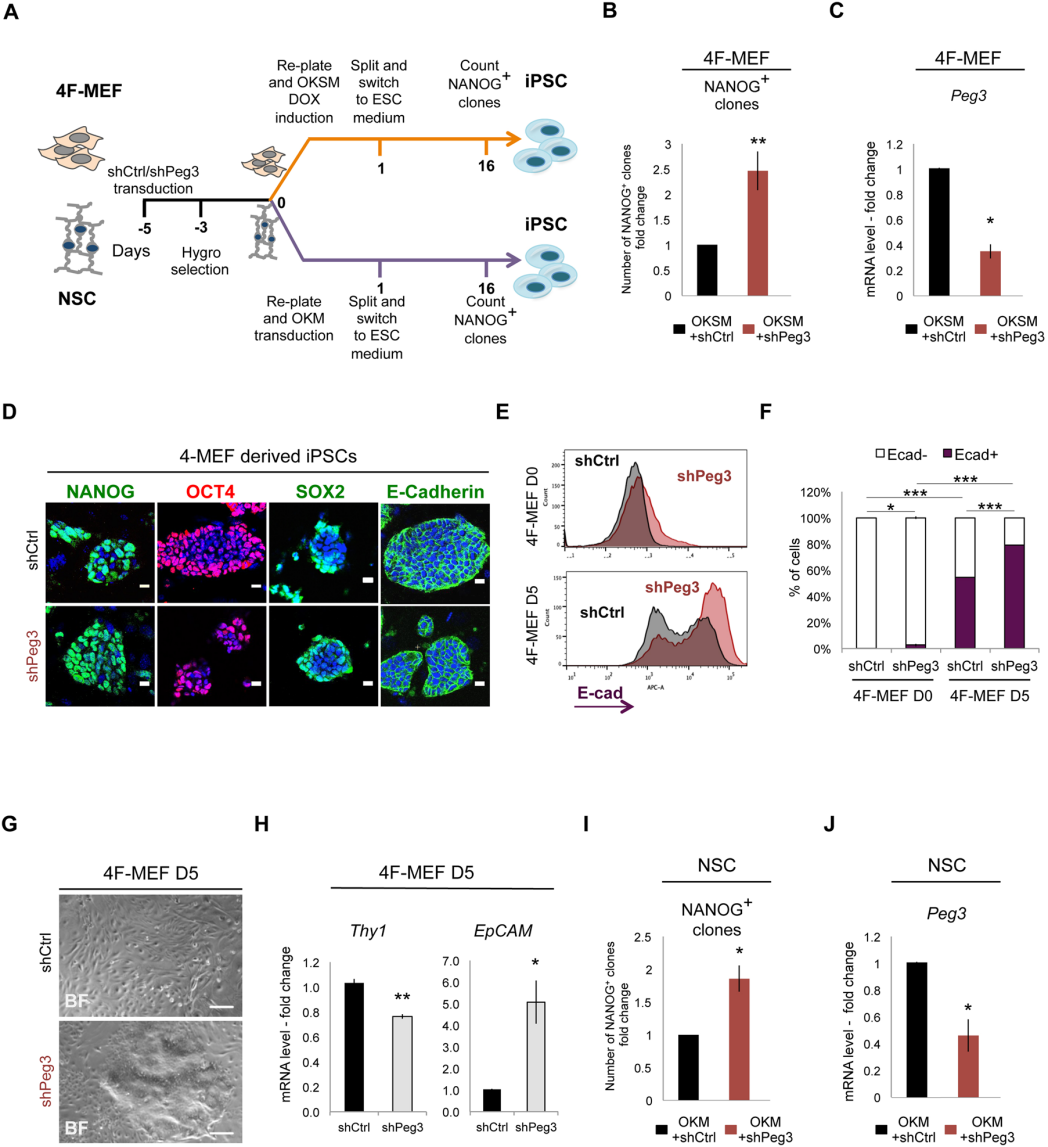
Peg3 silencing enhances MEF and NSC reprogramming efficiency. (**A**) Experimental scheme of MEF and NSC reprogramming infected with either shCtrl or shPeg3. MEFs and NSCs were infected with either shCtrl or shPeg3, hygromycin selected and re-plated in equal number before inducing reprogramming. The number of NANOG^+^ iPSC clones was counted at day 16 of reprogramming. (**B**) Number of NANOG^+^ iPSC clones (represented as fold change) obtained from shCtrl and shPeg3 infected 4F-MEFs (n = 3 independent experiments). (**C**) Quantitative real-time PCR analysis represented as fold change showing *Peg3* silencing efficiency at day 5 of MEF reprogramming (n = 3 independent experiments) (**D**) Representative fluorescence microscopy images of shCtrl and shPeg3 transduced iPSC clones expressing the pluripotency markers, such as NANOG, OCT4, SOX2 and E-cadherin. (**E,F**) Representative FACS profile showing E-cadherin expression in shCtrl and shPeg3 infected 4F-MEFs at day 0 (upper plot) and 5 (lower plot) of reprogramming (**E**) and its quantification (**F**). Data are represented as means of n = 3 independent experiments ± SD. (**G**) Representative bright field images of 4F-MEFs at day 5 of reprogramming infected with either shCtrl (upper) or shPeg3 (lower). (**H**) Quantitative real-time PCR analysis represented as fold change, showing *Thy1* and *EpCAM* expression (represented ad fold change) in shCtrl and shPeg3 infected 4F-MEFs at day 5 of reprogramming. (**I**) Number of NANOG^+^ iPSC clones (represented as fold change and n = 3 independent experiments) obtained from shCtrl and shPeg3 infected NSCs. (**J**) Quantitative real-time PCR analysis represented as fold change showing *Peg3* silencing efficiency at day 5 of NSC reprogramming. (**B**,**C**,**H**,**I**,**J**) Data are represented as fold change and means of n = 3 independent experiments ± SE. Asterisks indicate statistical significance calculated by unpaired two-tailed t test analysis (*p < 0.05; **p < 0.01; ***p < 0.001). Nuclei were stained with DAPI. Scale bar is 20 μm (**D**), 200 μm (**G**).

Based on the observations made in ESCs, we asked whether Peg3 could control the reversion of differentiated cells back to the pluripotent state. To address this hypothesis we decided to investigate the effect of *Peg3* KD during induced pluripotent stem cell (iPSC) generation starting both from MEFs and NSCs (Fig. 2A). First, we infected transgenic 4F-MEFs, obtained from the “reprogrammable” mice carrying the *Oct4*, *Sox2*, *Klf4*, *c-Myc* (OKSM) doxycycline (DOX) inducible cassette^28^ with either pLKO-shCtrl or pLKO-shPeg3 vectors, as previously described^25^. Subsequently we induced OKSM expression and we evaluated reprogramming efficiency by counting the number of NANOG positive (NANOG^+^) iPSC clones at day 16 (Fig. 2A). Interestingly *Peg3* KD enhanced MEF reprogramming process resulting in a higher number of generated NANOG^+^ clones when compared to the control (Fig. 2B). Importantly, by analyzing *Peg3* expression levels at day 5 of reprogramming we confirmed that the silencing was efficient also after OKSM induction (Fig. 2C). The generated iPSC clones obtained from both shCtrl and shPeg3 infected 4F-MEFs expressed different pluripotency markers, including NANOG, OCT4, SOX2 and E-cadherin (Fig. 2D). To then address if Peg3 can enhance the kinetic of iPSC generation, we performed FACS analysis for E-cadherin expression at day 0 and 5 of reprogramming. E-cadherin is an early reprogramming marker and its expression is detected upon cellular aggregation and mesenchymal-to-epithelial transition (MET) that occurs at the initial reprogramming stages^25, 29, 30^. Remarkably, shPeg3 transduced 4F-MEFs expressed higher levels of E-cadherin already at day 0 with respect to control (shCtrl), as shown by the increase of percentage of E-cadherin^+^ cells (Fig. 2E and F). During reprogramming E-cadherin expression gradually increased in both conditions, however its expression was significantly higher in Peg3 depleted 4F-MEFs when compared to the control (Fig. 2E and F). As a consequence, Peg3 depleted 4F-MEFs aggregated faster when compared to the control, as shown in Fig. 2G. Interestingly, *Peg3* inhibition was able to induce E-cadherin expression also in the absence of *c-Myc*. Indeed, OKS infected MEFs showed an increase in the percentage of E-cadherin^+^ cells at day 0 and at day 12 of reprogramming in the absence of *Peg3* (Supplementary Figure 2A and B). In absence of c-Myc, however, the reprogramming process was delayed and was less efficient, when compared to the OKSM induction, as indicated by the lower number of E-cadherin^+^ cells during reprograming (Fig. 2F and Supplementary Figure 2B), consistent with previously reported data^31^. These results suggest that *Peg3* KD has a positive effect on reprogramming and enhances its efficiency both in the presence and absence of *c-Myc*. However, we also observed that *Peg3* silencing alone is not sufficient to replace the effect of *c-Myc*.

To further strengthen our data, we also analyzed the expression levels of *Thy1* and *EpCAM*, mesenchymal and epithelial marker, respectively^25, 30^, again at day 5 of reprogramming. As expected, *Peg3* inhibition resulted in lower levels of *Thy1* and higher expression of *EpCAM*, suggesting that Peg3 could act already at the reprogramming onset (Fig. 2H).

Moreover, to investigate whether the effect of *Peg3* KD on reprogramming could be extended to other cell types, we also infected NSCs with either pLKO-shCtrl or pLKO-shPeg3 constructs and we induced reprogramming by overexpressing 3 factors, OCT4, KLF4, c-MYC (OKM), since NSCs already express high level of endogenous SOX2^32^ (Fig. 2A). As in the case of the MEFs, we observed more than two fold increase in the number of generated NANOG^+^ positive clones (Fig. 2I and Supplementary Figure 2C) upon *Peg3* silencing (Fig. 2J).

The effect of Peg3 KD was also tested by using the lentiviral-based miR30 inducible silencing system (pINDUCER10, miR-RUP) harboring a different Peg3 shRNA activated upon DOX addition. This construct also contained a constitutive puromycin resistance cassette and an RFP coding sequence, expressed along with the shRNA upon DOX treatment^33^ (Supplementary Figure 2D). Thus, we transduced E14 ESCs with the inducible Peg3 KD lentiviral construct (pIND-shPeg3), selected the cells with puromycin and analyzed the RFP expression both before and after DOX addition. Almost 90% of the infected cells were RFP positive (RFP^+^) already at 48 hours after DOX addition (Supplementary Figure 2E and F) and they showed an increase in pluripotency gene expression upon *Peg3* silencing (Supplementary Figure 2G). Moreover, the pIND-shPeg3 DOX-dependent silencing along with either OKSM or OKM expression (Supplementary Figure 2H) increased the number of NANOG^+^ iPSC clones (Supplementary Figure 2I and J), (around 5 folds) starting from both wild type (WT) MEFs and NSCs (Supplementary Figure 2K and L). *Peg3* silencing efficiency during reprogramming was around 50% at day 5 (Supplementary Figure 2M and N).

In conclusion, these data show that inhibition of Peg3 induces upregulation of pluripotency genes in ESCs and it enhances the efficiency of the reprogramming process starting from both MEFs and NSCs.

### PEG3 overexpression impairs somatic cell reprogramming

**Figure 3:**
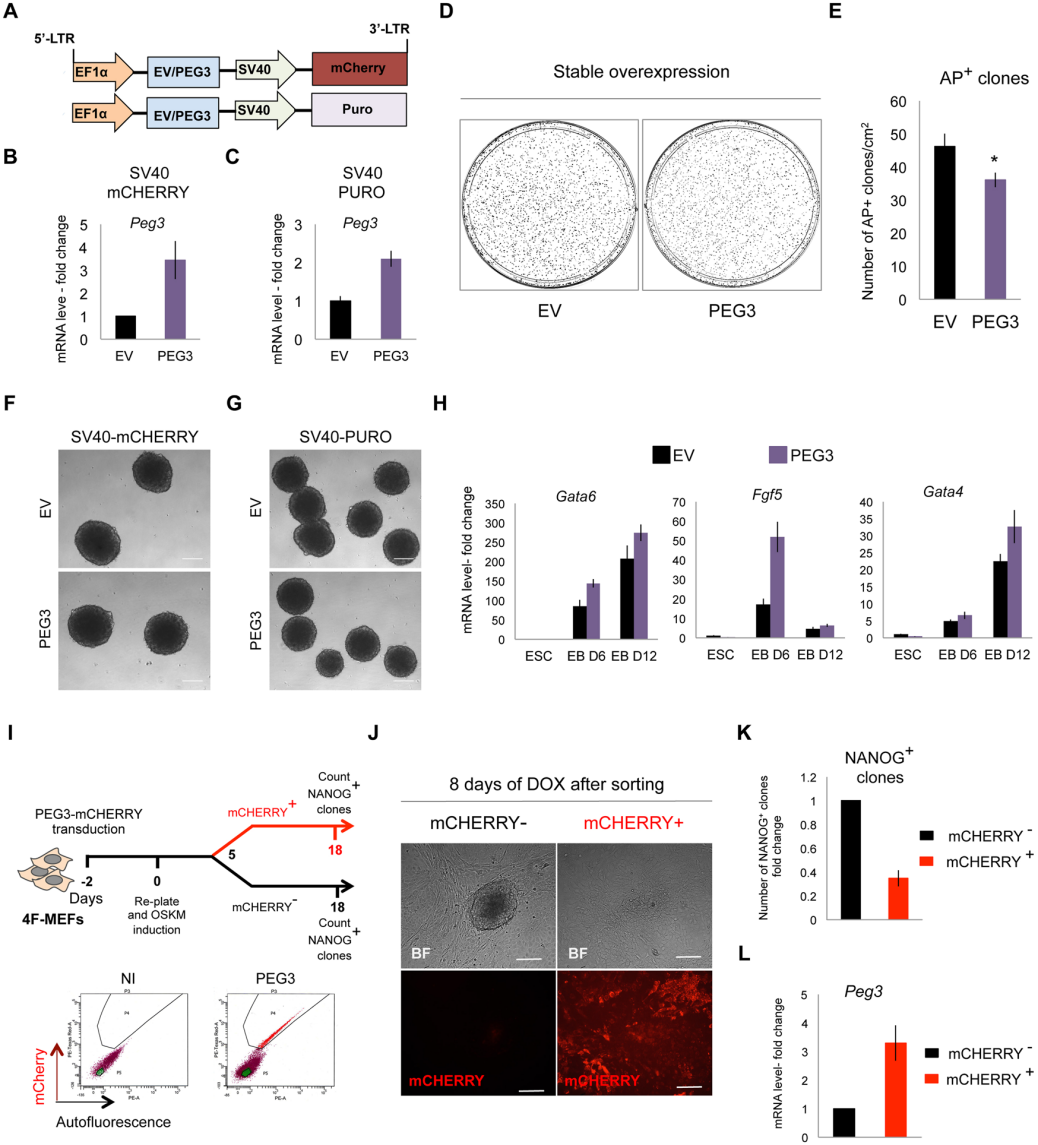
Peg3 overexpression impairs somatic cell reprogramming. (**A**) Representative scheme of EF1α-Peg3-SV40-mCherry and EF1α-Peg3-SV40-Puro lentiviral vectors. (**B,C**) Quantitative real-time PCR analysis of *Peg3* expression level after either EV or PEG3 transduction by using either EF1α-EV/Peg3-SV40-mCHERRY (**B**) (n = 3 independent experiments) or -PURO (**C**) (n = 3 technical replicates) (**D**) Representative AP^+^-CFC assay of ESCs transduced either with EF1α-EV-SV40-mCherry or EF1α-Peg3-SV40-mCherry, plated in equal number (10^2^ cells/cm^2^ for each condition) and cultured for additional 7 days. (**E**) Quantification of AP^+^ colonies at day 7 of AP^+^-CFC assay. Data are represented as means of n = 3 independent experiments ± SE. (**F**,**G**) Representative bright field images of EV and PEG3 infected EBs at day 5 of the differentiation protocol transduced with either EF1α-EV/Peg3-SV40-mCherry (**F**) or EF1α-EV/Peg3-SV40-Puro construct (**G**). (**H**) Quantitative real-time PCR showing differentiation markers expression levels (n = 3 technical replicates). Data are represented as fold change (2^−ΔΔCt^) and means ± SD. (**I**) Experimental scheme and plots showing FACS-sorting strategy of mCHERRY^+^ and mCHERRY^−^ MEFs at day 5 of reprogramming. The non infected (NI) MEFs were used as a control. At day 5 of reprogramming an equal number of mCHERRY^+^ and mCHERRY^−^ cells (8 × 10^3^ cells/cm^2^ for each condition) were FACS-sorted and re-plated on feeder layer in the presence of ESCs media + doxycycline (DOX). (**J**) Representative bright field images and fluorescence microscopy images, showing mCHERRY signal, of FACS-sorted mCHERRY^+^ and mCHERRY^−^ cells after additional 8 days of DOX treatment. (**K**) Number of NANOG^+^ iPSC clones (represented as fold change and mean of n = 2 independent experiments) counted at day 18 of DOX treatment after re-plating. (**L**) Quantitative real-time PCR showing *Peg3* expression levels (n = 2 independent experiments). (**B**,**C**,**K**,**L**) Data are represented as fold change (2^−ΔΔCt^) and means ± SE. Asterisks indicate statistical significance calculated by unpaired two-tailed t test analysis (*p < 0.05). Scale bar is 200 μm (**F**,**G**,**J**).

We asked whether Peg3 overexpression in ESCs and during iPSC generation process could perturb pluripotency and somatic cell reprogramming efficiency, respectively. To this aim, *Peg3* coding sequence was cloned under the constitutive EF1α promoter in a lentiviral vector carrying either a mCherry (EF1α-Peg3-SV40-mCherry) or a puromycin resistance cassette (EF1α-Peg3-SV40-PURO) driven by the constitutive SV40 promoter that allowed us to detect or select infected cells. In parallel, we also used an empty lentiviral vector (EV) as a control (Fig. 3A). After stable expression of either EF1α-Peg3-SV40-mCherry or EF1α-Peg3-SV40-PURO in ESCs we did not detect a strong upregulation of *Peg3*, which increased around 2-3 fold with respect to the control (EV) (Fig. 3B and C). Nevertheless, when we FACS-sorted the mCHERRY positive (mCHERRY^+^) ESCs and re-plated them in equal number to perform AP^+^-CFC assay we observed a reduction in the AP signal intensity and in the number of AP^+^ colonies after Peg3 overexpression when compared to the control (EV) (Fig. 3D and E). In addition, we performed transient Peg3 overexpression to further investigate its effect. In this case we used the EF1α-Peg3-SV40-mCherry construct in parallel with the EF1α-EV-SV40-mCherry vector (Supplementary Figure 3A), which allowed us to evaluate the efficiency of transfection by analyzing the FACS profile of the mCHERRY^+^ population. The transfection efficiency was comparable between EV and PEG3 overexpressing ESCs (Supplementary Figure 3B) and we further confirmed the PEG3 upregulation by analyzing the protein levels (Supplementary Figure 3C). Interestingly, by performing AP^+^-CFC assay we observed a decrease in the number of AP^+^ colonies along with high PEG3 levels (Supplementary Figure 3D and E), consistent with the phenotype observed in the stable Peg3 expression experiments.

Peg3 has been shown to be involved in cell apoptosis, acting together with p53^8, 34^. However, here, we excluded this possibility by performing AnnexinV (AnxV) staining and flow cytometry analysis (Supplementary Figure 3F and G). Indeed, within the mCHERRY^+^ population the number of AnxV positive cells remained very low in both EV and PEG3 overexpressing cells (around 1–2%).

Next, to investigate whether Peg3 overexpression could affect differentiation, we induced EB formation from ESCs overexpressing either EF1α-Peg3-SV40-mCherry (Fig. 3F) or EF1α-Peg3-SV40-PURO (Fig. 3G). In both cases we observed normal EB morphology. Nevertheless, the differentiation markers (*Gata6*, *Fgf5*, *Gata4*) corresponding to the three germ layers were higher in the EBs derived from EF1α-Peg3-SV40-mCherry overexpressing ESCs, when compared to the control (EV) (Fig. 3H). These data suggest that Peg3 overexpression confers ESCs a higher propensity to differentiate.

We, then, investigated if Peg3 overexpression could impair reprogramming. To this aim, we infected 4F-MEFs with the EF1α-Peg3-SV40-mCherry lentiviral construct and induced OKSM expression through DOX addition. At day 5 of reprogramming we FACS-sorted either mCHERRY^+^ or mCHERRY^−^ cells to enrich for both populations (with or without PEG3 overexpression) (Fig. 3I) and re-plated them in equal number on mitomycin C inactivated MEFs. Interestingly, after 8 days of DOX treatment since FACS-sorting, some iPSC clones started to appear only in the mCHERRY^−^ cell population. On the contrary the mCHERRY^+^ cells needed additional days to be reprogrammed, and iPSC colonies were detected only after 5 extra days of DOX treatment (Fig. 3J). Therefore, at day 18 the total number of NANOG^+^ clones derived from the mCHERRY^+^ population was much lower if compared to the mCHERRY^−^ derived cells (Fig. 3K). As expected the mCHERRY^+^ cells showed higher level of Peg3 (around 3-fold) when compared to the mCHERRY^−^ cells (Fig. 3L). These data suggest that high levels of Peg3 are detrimental for the reprogramming process.

### Peg3 regulates mitochondria mass and function during somatic cell reprogramming

Pluripotent stem cells are normally characterized by low OXPHOS that increases with differentiation^15–19^. Moreover, a metabolic switch from OXPHOS toward glycolysis, which is associated with the reduction of mitochondrial activity and increased cell proliferation, is indispensable for the iPSC generation process^14, 20, 35–39^. Interestingly, Peg3 has been previously described to bind to genes that control mitochondrial functions, (*Ndufs7*, *Ndufs8*, *Sdhb)* and tissue development^11, 12^. In particular, Peg3 can recognize specific binding sites in the promoter regions through a specific consensus DNA-binding motif and its depletion can affect the expression of the corresponding genes^11^. During somatic cell reprogramming *Ndufs8* and *Sdhb* expression has been associated with increased mitochondrial mass, cell size and low number of generated iPSCs^37^. Based on previously reported data, we asked whether Peg3 perturbation could alter mitochondrial gene expression and mitochondrial activity in both pluripotent stem cells and during somatic cell reprogramming.

**Figure 4:**
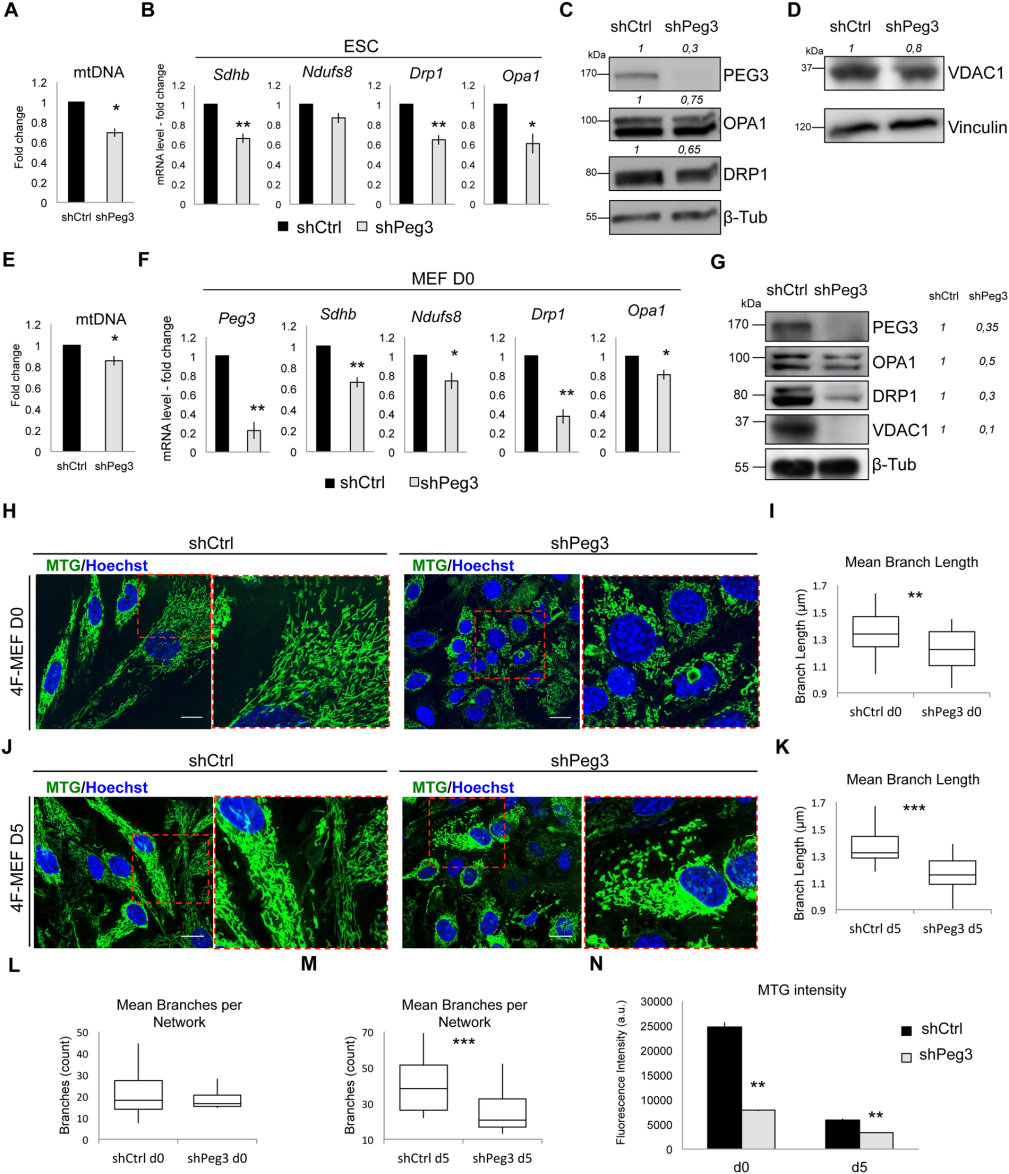
Peg3 silencing causes mitochondrial impairment. (**A**) Quantitative real-time PCR analysis showing mitochondrial DNA quantification in ESCs normalized over *Gapdh* genomic DNA region **(B**). Quantitative real-time PCR analysis of mitochondrial gene expression levels in ESCs. (**C,D**) Representative western-blots (out of n = 2 independent experiments) showing PEG3 silencing efficiency and the level of mitochondrial proteins such as OPA1, DRP1 (**C**) and VDAC1 (**D**) in shCtrl and shPeg3 transduced ESCs. (**E)** Quantitative real-time PCR analysis showing mitochondrial DNA quantification in MEFs normalized over *Gapdh* genomic DNA region. (**F**) Quantitative real-time PCR analysis of mitochondrial gene expression levels in MEFs. (**G**) Representative western-blot (out of n = 2 independent experiments) showing PEG3 silencing efficiency and the level of mitochondrial proteins such as OPA1, DRP1 and VDAC1 in MEFs at day 0 after shCtrl and shPeg3 infection. (**H,J**) Representative fluorescence microscopy images and higher magnification of the selected area of MEFs at day 0 (**H**) and 5 (**J**) of reprogramming transduced with either shCtrl or shPeg3, showing mitochondria morphology stained with MitoTracker Green (MTG). Scale bar is 20 μm and nuclei are stained with Hoechst 33342. (**I,K**) Box plot representing mean mitochondrial branch length quantification (corresponding to the average length of all branches) in shCtrl and shPeg3 infected MEFs and day 0 (**I**) and day 5 (**K**) of reprogramming. (**L**,**M**) Box plot representing mean mitochondrial network size quantification (corresponding to the number of branches per network) in shCtrl and shPeg3 infected MEFs and day 0 (**L**) and day 5 (**M**). (**N**) Quantification of MTG intensity from day 0 to day 5 of reprogramming in MEFs transduced either with shCtrl or shPeg3. (**A**,**B**,**E**,**F,N**) Data are represented as fold change (2^−ΔΔCt^) and means ± SE of n = 3 independent experiments. Asterisks indicate statistical significance calculated by unpaired two-tailed t test analysis (*p < 0.05; **p < 0.01; ***p < 0.001). For western-blot analysis β-Tubulin and Vinculin were used as loading controls and densitometric analysis was carried out by using ImageJ software. The quantification reflects the relative amounts as a ratio of each protein band relative to their loading control.

By measuring both mitochondrial DNA amount and expression of mitochondrial-related genes, we observed a general downregulation in Peg3 depleted ESCs, as compared to the control (Fig. 4A and B). However, this was translated only into a minor decrease in the levels of proteins that control mitochondrial dynamics (such as OPA1 and DRP1^40^) and of proteins of the outer mitochondrial membrane, such as VDAC1^41^ (Fig. 4C and D). In parallel, we also analyzed mitochondrial potential by performing FACS-analysis of tetramethylrhodamine ethyl ester (TMRE) intensity^16, 42^. This showed only a minor, not significant, decrease after Peg3 silencing (Supplementary Figure 4A). Perhaps, this poor effect was due to the fact the Peg3 endogenous level was very low in ESCs and its further downregulation did not induce major changes in the mitochondrial activity. As control, we ensured that TMRE intensity was strongly and significantly downregulated after 16 h treatment of ESCs with the un-coupler carbonyl cyanide-m-chlorophenylhydrazone (CCCP)^43^, but not with ethanol (EtOH) (Supplementary Figure 4B), indicating good specificity and sensitivity of the TMRE assay. To further investigate the correlation between *Peg3* levels and mitochondrial activity in ESCs we analyzed mitochondrial gene expression during EB differentiation of Peg3 depleted ESCs. As expected, both shCtrl and shPeg3 infected ESCs could form normal EB structure (Supplementary Figure 4C). However, the expression levels of differentiation markers *Fgf5* and *Gata4* (markers of ecto- and mesoderm layer, respectively), were lower in the Peg3 depleted EBs when compared to the control (Supplementary Figure 4D). Interestingly, also mitochondria gene expression followed *Peg3* expression during EB differentiation, suggesting a delay in differentiation in ESCs carrying Peg3 KD (Supplementary Figure 4E).

Since Peg3 KD only showed a mild phenotype in ESCs we asked whether its downregulation was effective in MEFs and NSCs that both express high endogenous *Peg3* levels. First, we analyzed mitochondrial DNA amount and mitochondrial related gene expression in MEFs carrying either pLKO-shCtrl or pLKO-shPeg3 silencing constructs. These were significantly downregulated after *Peg3* inhibition (Fig. 4E and F). Moreover, mitochondrial proteins (OPA1, DRP1, VDAC1) showed drastic reduced levels in shPeg3 infected MEFs as compared to control (Fig. 4G). Interestingly, MEFs carrying Peg3 KD displayed smaller cell size and less elongated mitochondria both at day 0 and at day 5 of reprogramming (Fig. 4H and J), suggesting that *Peg3* inhibition could induce mitochondria structural remodeling. To further characterize the effect of Peg3 inhibition on mitochondria morphology we quantified mitochondrial branch length by using Mitochondrial Network analysis (MiNA) toolset^44^ (Fig. 4I and K and Supplementary Figure 4F and G). Indeed, both mean and median branch length were significantly lower in Peg3 depleted MEFs at day 0 (Fig. 4I and Supplementary Figure 4F) and day 5 of reprogramming (Fig. 4K and Supplementary Figure 4G). Additionally, the mean mitochondrial network size, which indicates the number of branches per network^44^, was again lower in shPeg3 infected MEFs with respect to the control (Fig. 4L and M). These results indicate that Peg3 inhibition correlates with less elongated and less branched mitochondria in MEFs, both at day 0 and 5 of reprogramming. Furthermore, at day 0 of reprogramming both mitochondrial mass and potential, according to the FACS-analysis profile of MitoTracker Green (MTG) and TMRE respectively^43, 45, 46^, were lower upon Peg3 silencing with the respect to the control (Fig. 4N and Supplementary Figure 4H–L). In line with previously reported data^37^, the intensity of MTG and TMRE decreased during reprogramming from day 0 to day 5 in both conditions; however it was maintained even lower in Peg3 depleted MEFs (Fig. 4N and Supplementary Figure 4H–L). All in all, these data suggest that Peg3 inhibition induces mitochondria morphology remodeling during somatic cell reprogramming.

**Figure 5:**
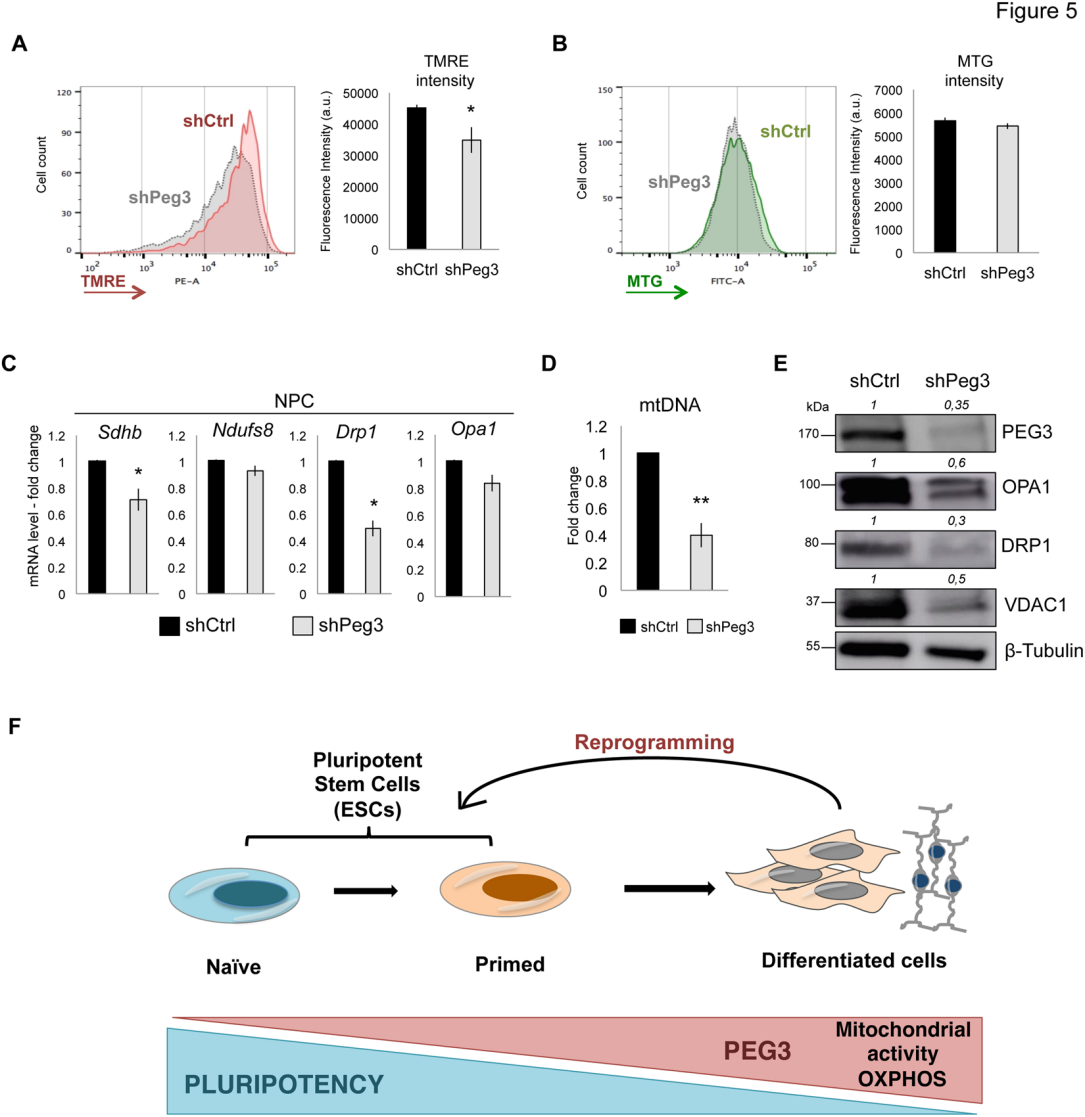
Peg3 levels correlate with mitochondrial mass and activity. (**A**,**B**) Representative FACS plot analysis and quantification of tetradmethylrhodamine ethyl ester (TMRE) (**A**) and MTG (**B**) intensity in NSCs transduced with either shCtrl or shPeg3 and selected with hygromycin (n = 3 independent experiments). (**C**) Quantitative real-time PCR analysis of mitochondrial gene expression levels in NSCs represented as fold change (2^−ΔΔCt^) and means ± SE of n = 3 independent experiments. (**D**) Quantitative real-time PCR analysis showing mitochondrial DNA quantification in NSCs calculated over *Gapdh* genomic DNA region (n = 3 independent experiments). (**E**) Representative western-blot (n = 1) showing PEG3 silencing efficiency and the levels of mitochondrial proteins such as OPA1, DRP1, and VDAC1 in shCtrl or shPeg3 transduced NSCs. (**F**) Schematic representation explaining the role of Peg3 in pluripotent stem cells and during somatic cell reprogramming. Asterisks indicate statistical significance calculated by unpaired two-tailed t test analysis (*p < 0.05; **p < 0.01). For western-blot analysis β-Tubulin was used as loading control and densitometric analysis was carried out by using ImageJ software. The quantification reflects the relative amounts as a ratio of each protein band relative to their loading control.

Peg3 silencing induced reduction of TMRE intensity also in NSCs (Fig. 5A), although the mitochondrial mass did not decrease in this case, as shown by the MTG intensity (Fig. 5B). As in the case of MEFs, Peg3 silencing correlated with lower mitochondrial related gene expression and mitochondrial DNA levels (Fig. 5C,D and E). These observations indicate that Peg3 KD causes an overall mitochondrial impairment in both MEFs and NSCs.

To further explore the correlation between Peg3 levels and mitochondrial activity we transfected ESCs with either EF1α-EV-SV40-mCherry or EF1α-Peg3-SV40-mCherry and we measured MTG intensity within the mCHERRY positive population in each condition. PEG3 overexpressing ESCs (Supplementary Figure 5A) displayed higher MTG intensity when compared to the control (EV) (Supplementary Figure 5B) and higher levels of outer mitochondrial membrane protein, VDAC1 (Supplementary Figure 5C). Interestingly, PEG3 overexpression also correlates with increased protein levels of the mitochondrial oxidative complexes. In particular, ATP synthase subunits, corresponding to complex 5 (CV- ATP5A) and ubiquinol-cytochrome c reductase subunits corresponding to complex 3 (CIII-UQCRC2) were upregulated upon PEG3 overexpression (Supplementary Figure 5D). In line with these results, Peg3 overexpression increased the protein levels of the succinate dehydrogenase complex subunit A (SDHA), the iron-sulfur subunit (SDHB) (Supplementary Figure 5E) and mitochondrial DNA amount (Supplementary Figure 5F) in ESCs.

Finally, to strengthen our hypothesis of Peg3 controlling mitochondrial activity we also transfected HEK293T cells with either EF1α-EV-SV40-mCherry or EF1α-Peg3-SV40-mCherry constructs and analyzed MTG intensity level within the mCHERRY positive cell population. Also in this case, the mCHERRY^+^ cells overexpressing PEG3 (Supplementary Figure 5G) displayed higher mitochondrial mass with the respect to the mCHERRY^+^ overexpressing an empty vector (EV), as shown by MTG intensity levels (Supplementary Figure 5H).

All in all, these data suggest that Peg3 could regulate mitochondrial structure, mass and activity within different cell types. Moreover, perturbation of Peg3 expression levels affect the efficiency of somatic cell reprogramming process, which correlates with the mitochondria undergoing structural and functional modifications, leading to metabolic changes.

## Discussion

Peg3/Pw1 is widely expressed during embryonic and fetal development and in particular in the muscle, neuronal lineages and placenta^1, 2, 47^. Moreover, high Peg3 levels have been observed in many adult tissues such as brain, ovary, testis and in adult stem cells^3, 48^. In this study we observed that Peg3 is expressed at very low levels in ESCs, while it is upregulated during ESC differentiation towards NSCs or in embryoid bodies. These results are consistent with the published observation that Peg3 starts to be robustly expressed in the mouse embryo at the beginning of the gastrulation during muscle development and neurogenesis^1, 2^.

Although ESCs showed low endogenous Peg3 expression when grown in serum-Lif medium, a further transcriptional downregulation was observed in 2i-Lif cell culturing condition. ESCs cultured in 2i-Lif medium have been shown to be more similar to the inner cell mass^24^, while ESCs cultured in serum-Lif medium display heterogeneous pluripotency marker expression and are subject to spontaneous differentiation events^49^. This difference might explain the distinct levels of Peg3 in these two ESC pluripotent states. Interestingly, *Peg3* knockdown in ESCs cultured in serum-Lif led to an increase of pluripotency marker expression and AP^+^ signal, although Peg3- silenced cells did not reach the ground state pluripotency that ESCs reach when grown in the 2i-Lif medium. Indeed, REX1-dGFP ESCs are almost all GFP positive when cultured in 2i-Lif medium^23^. This was not observed in the case of Peg3 depleted ESCs. However, Peg3 depleted ESCs showed a delay in differentiation toward NSCs, suggesting that Peg3 KD might prevent differentiation and enhance pluripotency, even though it was not sufficient to allow the cells to reach the ground state pluripotency. Additionally, Peg3 silencing caused a delay in the ecto- and mesodermal marker expression during EB differentiation. On the contrary these differentiation markers were upregulated in PEG3 overexpressing ESCs. These results suggest that Peg3 plays an important role in cell fate determination, which further confirm previous reported data showing *Peg3* expression in muscle and neural lineage formation during embryo development^1, 2^.

In addition to their differentiation capability, pluripotent stem cells are characterized by a high proliferative capacity that normally correlates with elevated glycolytic metabolism and reduction of oxidative phosphorylation (OXPHOS) within the mitochondrial complexes^18, 19, 36, 50–53^. The glycolytic flux decreases dramatically when ESCs start to differentiate^15, 54–56^ and, in parallel, mitochondrial OXPHOS increases. For instance, the switch from glycolysis to mitochondrial oxidative metabolism is characterized also by mitochondria maturation and it is necessary to allow cardiac specification^16, 54^. These published observations are again consistent with our data, which show a strong and significant increase of *Peg3* expression during EB formation. Therefore *Peg3* expression correlates with high oxidative metabolism that is necessary to boost cell fate transition. In line with these results, *Peg3* has been reported to mediate muscle stem cell homeostasis^57^. Interestingly, several cancer cell lines, including glioma, have been shown to downregulate *Peg3* ^6^. Similar to ESCs, glioma stem cells and many cancer cells are characterized by high glycolytic metabolism^58^, confirming our observation that *Peg3* expression can be associated to OXPHOS metabolism rather than glycolysis.

A switch from OXPHOS to glycolysis has been shown to occur during reprogramming of somatic cells into iPSCs^20, 35^, preceding the reactivation of pluripotency markers^59^ and it is associated with rapidly proliferating cells. Moreover promotion of glycolysis or stimulation of OXPHOS can enhance or impair somatic cell reprogramming process, respectively^20, 35^. In a similar way, in our study we observed that Peg3 silencing could enhance the yield of iPSC generation starting from either NSCs or MEFs, suggesting that its effect controls the dynamics of the reprogramming process and, importantly, it is not cell type specific. This is supported by the finding that even a modest Peg3 overexpression impairs the generation of NANOG^+^ iPSC clones. In particular, we observed that Peg3 silencing caused a generalised mitochondrial impairment, as shown by the decrease of MTG and TMRE intensity and mitochondrial gene expression downregulation, such as *Ndufs8*, *Sdhb*, *Drp1* and *Opa1*. This phenotype was particularly evident in MEFs at day 0 and during the reprogramming process. Consistently, Peg3 has been shown to bind directly to *Ndufs7*, *Ndufs8*, *Sdhb* and other mitochondrial genes in a sequence specific manner, behaving, therefore, as a DNA-binding protein^11, 12^. These nuclear encoded mitochondrial genes are involved in the maintenance of energy homeostasis, further supporting the connection between Peg3 and mitochondrial activity. Interestingly, we observed that *Ndufs8*, *Sdhb* and *Peg3* expression levels correlated in ESCs, NSCs and MEFs suggesting that Peg3 can control common genes in these different cell types. *Ndufs8* and *Sdhb* encode for subunits of the NADH-ubiquinone oxidoreductase complex and the succinate dehydrogenase complex, respectively. These proteins are located within the mitochondria membrane and they regulate OXPHOS^60, 61^.

Additionally, we observed that Peg3 silencing caused a decrease in mitochondrial mass, as compared to the control, suggesting a possible reduction also in OXPHOS. Accordingly, Peg3 depleted MEFs displayed less branched and elongated mitochondria during somatic cell reprogramming. Indeed, the remodeling of the mitochondria structure^62^ and the reduction of mitochondrial mass^37^ was previously associated with the reprogramming process. Moreover, we observed the opposite phenotype following *Peg3* overexpression, which also correlates with upregulation of the OXPHOS protein complexes, thus further confirming its effect on mitochondria. This role of Peg3 in the regulation of metabolic changes could be extended to other cell types and tissues. Previous published data report that Peg3 can regulate mammalian growth and behavior^63^. In particular, *Peg3* expression has been detected in the embryo and adult brain and mutations in the *Peg3* gene cause behavioral defects, such as impairment of maternal behavior that could be caused by defective neuronal connectivity^63^. Mature neurons rely on oxidative phosphorylation to meet high-energy demands; consequently defects in mitochondria structure and metabolism are often associated with neurodegenerative disorders^64–66^. Moreover other *in vivo* models further support the involvement of Peg3 in metabolic regulation and our observations: for example, mice carrying a targeted mutation in *Peg3* gene displayed increased body fat^67^. Finally, downregulation of *Peg3* and other paternally expressed genes in adipocytes has been shown to be involved in the paternal transmission of high-fat-diet induced obesity^68^.

Overall our study demonstrates that *Peg3* expression levels anti-correlate with pluripotency and increase gradually upon ESC differentiation. Moreover, downregulation of *Peg3* enhances the efficiency of reprogramming of differentiated cells towards pluripotency. In particular, Peg3 can alter mitochondrial activity, which could contribute to the OXPHOS-to-glycolysis metabolic switch (Fig. 5F). Therefore Peg3 can be regarded as a new and important player of metabolic and molecular changes that in turn can control cell fate transition.

## Material and Methods

### iPSC induction and ESC differentiation

The reprogramming experiments of OKSM 4F-MEFs were routinely conducted in gelatin-coated plates, in ESC culture medium supplemented with 2 μg/ml doxycycline, as previously described^28^. For WT MEFs reprogramming was induced through OKSM or OKS infection. NSCs were reprogrammed through OKM overexpression. In particular, 2 × 10^5^ MEFs and NSCs were transduced with OKSM/OKS/OKM concentrated retroviral particles. After 48 h the infected cells were plated on feeder layer and the media was switched to ESC media for 16 additional days. Doxycycline was added up to day 12 of reprogramming. The number of reprogrammed iPSC clones was counted at day 16 of reprogramming.

### Mitochondria mass and potential quantification

Mitochondria were stained in living cells with MitoTraker Green (Invitrogen M7514) (150 nM) for 15 min at 37 °C and 5% CO_2_. MitoTraker Green incorporation was quantified by BD LSR Fortessa flow cytometer and analyzed by Flowjo software. Unstained cells were used as negative control and DAPI (SIGMA 09542) staining was performed to exclude dead cells from the analysis. Before FACS analysis the samples were filtered with 35μm mesh size filters (Corning Life Sciences 352235) to avoid aggregates.

Mitochondria potential was assessed by Mitostatus TMRE staining (BD Pharmingen 564696). Living cells were labeled with Mitostatus TMRE (100 nM) for 15 min at 37 °C and 5% CO_2_. Mitochondrial potential was quantified by BD LSR Fortessa flow cytometer and analysed by Flowjo software. Unstained cells were used as negative control and DAPI (SIGMA 09542) staining was performed to exclude dead cells from the analysis. Before FACS analysis the samples were filtered with 35μm mesh size filters to avoid aggregates.

## Electronic supplementary material

Supplementary information and figures
