# Supplementary material for: Reduced expression of Paternally Expressed Gene-3 enhances somatic cell reprogramming through mitochondrial activity perturbation
Source: Sci Rep. 2017 Aug 29;7:9705. doi: 10.1038/s41598-017-10016-7 (PMC5575273; doi:10.1038/s41598-017-10016-7)

Inventory of Supplementary Information

Reduced expression of Paternally Expressed Gene-3 enhances somatic cell reprogramming through mitochondrial activity perturbation

Ilda Theka, Francesco Sottile, Francesco Aulicino, Alvaro Castells Garcia and Maria Pia Cosma

1. Supplementary Materials and Methods
2. Supplementary References
3. Supplementary Figure Legends
4. Supplementary Figures

1. Supplementary Materials and Methods

Cell lines

E14 ESCs (129/Ola) or REX1-dGFP ESCs (129Sv) were maintained onto gelatin (Millipore ES-006-B)-coated plates with DMEM supplemented with 15% fetal bovine serum (FBS), L–glutamine (2mM), penicillin (100U/ml), streptomycin (100µg/ ml), sodium pyruvate (1mM), non-essential amino acid (NEAA) (0,1mM), 2-mercaptoethanol (0,5mM) and ESGRO mLif (1000U/ml). HEK293T cells were grown in DMEM supplemented with 10% fetal bovine serum (FBS), L– glutamine (2mM), penicillin (100U/ml), streptomycin (100µg/ ml), sodium pyruvate (1mM), non-essential amino acid (NEAA).

MEFs were established from E13.5 embryos either from WT C57BL/6J or from reprogrammable mice carrying two (ho/ho) copies of the OKSM inducible cassette and the ROSA26-M2rtTA allele [1]. The embryos were isolated from the uterus and washed in phosphate-buffered saline (PBS). The head and viscera were removed, and the rest of the body was mechanically disaggregated and then incubated in 0.1% trypsin/ 0.1 mM EDTA solution for 30 min, to allow the cells to detach from the extracellular matrix and from each other. The cells were then mechanically disaggregated and plated onto 15-cm tissue-culture dish and cultured in MEF culture medium: Dulbecco's modified Eagle's medium (DMEM) supplemented with 10% fetal bovine serum (FBS), L–glutamine (2mM), penicillin (100U/ml), streptomycin (100µg/ ml), sodium pyruvate (1mM), non-essential amino acid (NEAA). The MEFs were kept at 37°C under 5% CO_2.

NSC-GiP cells carrying the regulatory sequences of the mouse *Oct4* gene driving GFP and puromycin resistance were a gift from Dr. A. Smith. NS-GiP cells were

obtained from HP165 mice and maintained in Poly-l-Ornithine (SIGMA P4957) and Laminin (SIGMA L2020) coated plate and cultured in RHB-A supplemented with penicillin (100U/ml), streptomycin (100µg/ ml), bFGF (Peprotech AF-100-18B) (10ng/ml) and EGF (Peprotech AF-100-15C) (50ng/ml) as previously described [2,3].

NSC and EB differentiation

Prior to initiating monolayer differentiation, ESCs were plated in standard ESC medium containing LIF. After 24h, undifferentiated ESCs were gently dissociated using trypsin (0.025% trypsin and 0.04% EDTA) at 37°C and plated onto 0.1% gelatin-coated tissue culture plastic at a density of 0.5–1.5x10^4 /cm2 in N2B27 medium [1:1mix of DMEM/F12 (GIBCO) supplemented with N2 (GIBCO) and Neurobasal medium (GIBCO) supplemented with B27 (GIBCO)], L–glutamine (0,5 mM), 2-mercaptoethanol (0,1mM) and retinoic acid (1µM). The medium was refreshed every other day.

For embryoid body (EB) induction, the differentiation medium consisted of ESC culture medium without LIF. The cells were harvested by trypsinisation, counted, and propagated in hanging drops (400 single ESCs/ 30 µl initial drop) for 2 days, before being transferred to 10 cm^2 bacterial dishes. On day 5, embryoid bodies were transferred onto gelatin-coated 10 cm^2 tissue culture dishes always in differentiation medium.

Flow cytometry

For analysis and/or sorting of $GFP^{+/-}$ or $mCHERRY^{+/-}$ cell populations, cells were trypsinized, washed once in PBS, and resuspended in PBS with 5% FBS plus DAPI (SIGMA 09542). Untreated cells were used as negative staining control and DAPI staining was performed to exclude dead cells from the analysis. Before FACS analysis the samples were filtered with 35µm mesh size filters (Corning Life Sciences 352235) to avoid aggregates.

For apoptosis detection, phosphatidylserine exposure was detected by flow cytometry using APC Annexin V (BD pharmingen 550474) following the manufacturer's instructions, quantified using BD LSR Fortessa and analyzed by Flowjo software. Unstained cells were used as negative control.

For E-cadherin staining, cells were collected and incubated with Fc receptor blocking reagent (anti-Mouse CD16/CD32, eBioscience 14-0161-82) in PBS with 5% FBS for 10 min at 4°C. The cells were washed once in PBS plus 5% and incubated for 20 min at 4°C with E-cadherin antibody (0,5 $\mu g/10^6$ cells, Biolegend 147308) in PBS with 5% FBS and DAPI. E-cadherin expression was quantified by BD LSR Fortessa flow cytometer and analyzed by Flowjo software. Unstained cells were used as negative control and DAPI (SIGMA 09542) staining was performed to exclude dead cells from the analysis. Before FACS analysis the samples were filtered with 35µm mesh size filters (Corning Life Sciences 352235) to avoid aggregates.

Mitochondria live-cell imaging and morphological features quantification

Living cells labeled with MitoTraker Green were cultured onto Thermo Scientific Nunc Lab-Tek chambered coverglass (155411) and fluorescence images were

obtained using Leica TCS SP5 CFS confocal microscope. Nuclear staining was performed with Hoechst 33342 (ThermoFisher H1399).

Images processing was performed using Fiji software[4] and mitochondria morphological features were identified and characterized using MiNA macros as previously described[5]. MiNA software is freely available at https://github.com/ScienceToolkit/MiNA.

Constructs preparation

Short hairpins targeting Peg3 (shPeg3) and a short hairpin control (shCtrl) were cloned into the pLKO.1-Hygro lentiviral vector (Addgene plasmid #24150), following the manufacturer instructions (http://www.addgene.org/tools/ protocols/plko/). The oligonucleotides cloned into the pLKO vector were purchased from Sigma-Aldrich. To silence Oct4 we used the oligonucleotides published by Ang and colleagues [6]. The oligonucleotides used to generate the short hairpins are given in the Supplementary Table 1.

**Short hairpin**	**ENSEMBL Gene_ID**	**Top oligo sequence (5'-3')**
sh-Peg3 (CDS)	ENSMUSG 00000002265	CCGG**CCCTAATGACAAGCTGAAATT**CTCGAGAATTTCAGCTTGTCATTAGGGTTTTTG
sh-Ctr		CCGG**GTCACGATAAGACAATGAT**CTCGAGATCATTGTCTTATCGTGACTTTTT

Supplementary Table 1. List of top oligonucleotides used for cloning short-hairpins into pLKO-Hygro digested with AgeI/EcoRI. Sense target sequences are highlighted in bold.

The pIND-shPeg3 was subcloned from pGIPZ-shPeg3 (Dharmacon, Clone ID: V2LMM-50775) into the pINDUCER10 (miR-RUP) with XhoI and MluI [7]. Peg3 cDNA sequence was obtained from Dharmacon MGC collection (Clone ID 30543809, Accession BC072661) and it was cloned into the pL-EF1α vector containing either a constitutive puromycin or mCherry cassette. In order to generate lentiviral overexpression vectors, 7TGC and 7TGP reporter lentiviral plasmids (Addgene #24305; #24304) [8] were repurposed for gene expression. Briefly eGFP was removed by enzymatic digestion and 7xWRE/CMV-minimal promoter was replaced by a constitutive EF1α promoter from p1494 vector, kind gift of Luigi Naldini, followed by a multiple cloning site with a unique SmaI restriction site, suitable for blunt linearization and subsequent use for Gibson Assembly. eGFP was removed by 7TGP or 7TGC digestion with XbaI, plasmid was re-ligated to generate 7TP or 7TG intermediate vectors. 7TP or 7TG were digested with SmaI to create promoterless lentiviral vectors and substituted with EF1α promoter through Gibson Assembly. SmaI 3' site was maintained for subsequent cloning of Peg3 cDNA (MGC collection, Clone ID: 30543809; Accession: BC072661). Oligos for EF1a amplification and subcloning are provided below (Supplementary Table 2).

**Target**	**Template**	**Oligos (5'-3')**
**EF1α promoter**	**p1494 EF1a vector**	FW:ATCGACGGTACCGCG GGCCCTCCGCGGCCGCGG ATCCCCC<br><br>RV:GACTCTAGATCTCGAG CCCCAGCTTCTCGAGGAA TTCCCC

Supplementary Table 2. Oligos for EF1α amplification and subcloning.

Virus preparation and cell infection

For ESC infection, lentiviral particles were produced following the RNA interference Consortium (TRC) instructions for lentiviral particle production and infection in 6-well plates (http://www.broadinstitute.org/rnai/public/). Briefly, 5 $\times 10^5$ HEK293T cells/well were seeded in 6-well plates in DMEM supplied with 10% FBS, 10 u/ml penicillin, streptomycin 10μg/ml, 2 mM glutamine, 1mM sodium pyruvate and non-essential amino acids. The day after plating, the cells were co-transfected with 1 μg of each of the lentiviral plasmids (pLKO-shCtrl, pLKO-shPeg3, pIND-shPeg3, EF1α-Peg3-SV40-mCherry, EF1α-EV-SV40-mCherry, EF1α-EV-SV40-PURO, EF1α-Peg3-SV40-PURO), 750 μg pCMV-dR8.9, and 250 μg pCMV-VSV-G, using Polyfect reagent (Qiagen). The day after transfection, the HEK293T culture medium was substituted with the ESC culture medium. Then 5 $\times 10^4$ ESCs/well were plated onto gelatin-coated 6-well plates the day before transduction. The lentiviral-containing medium was harvested from HEK293T cells at 48 and 72 h after transfection, filtered, and added to the ESC plates. The day after transduction, the ESCs were washed twice in PBS and hygromycin selection (50 μg/ml) was applied.

For MEF and NSC reprogramming 2 $\times 10^5$ cells were infected with concentrated virus supernatant. Briefly 7,5 x 10^6 HEK293T cells were seeded on 15 cm dishes and incubated overnight. The following mix was prepared for the transfection: 32 μg of lentiviral plasmids (shPeg3, shCtrl, pIND-shPeg3, EF1α-Peg3-SV40-mCherry, EF1α-EV-SV40-mCherry) or retroviral plasmids (Oct4, Klf4, Sox2, c-MYC), 20 μg pCMV-dR8.9, 9 μg pCMV-VSV-G. Successively, 125 μl of CaCl2 2,5 M and 1,25 ml of

HBS, were added to the mix, while vortexing at full speed. The final mix was then dropped over the cells. After 48 and 72h hours the supernatant was collected and ultracentrifugated at 20.000 rpm for 2 hours at 20°C. The pellet with viruses was resuspended in PBS and stored at -80°C.

For transient silencing or overexpression experiments 5 $\times 10^4$ ESCs/well or HEK293T were plated onto gelatin-coated 6-well plates and transfected with Polyfect reagent (Qiagen) and 2 µg of each of pLKO-shCtrl, plKO-shPeg3, EF1α-EV-SV40-mCherry or EF1α-Peg3-SV40-mCherry plasmids following manufacturer's instruction.

Immunofluorescence staining

For immunocytochemistry, the cells were fixed with 4% paraformaldehyde for 20 min at room temperature, and then washed twice with PBS. These fixed cells were then incubated in blocking solution containing 10% goat serum (Sigma) and 0.1% Triton X-100 (Sigma) for 1h at room temperature. The cells were then left overnight at 4°C in blocking solution containing the primary antibody. The next day, the cells were washed three times with PBS and then incubated with the secondary antibody for 1h at room temperature. The primary antibody against rabbit NANOG (Calbiochem #SC1000), rabbit SOX2 (ab97959), mouse OCT3/4 (sc-5279), mouse E-cadherin (BD610182) were used, and the goat anti-rabbit, goat anti-mouse IgG, (1:1000, Life Technologies) conjugated to Alexa Fluor-488 and Alexa Fluor-568 were used as secondary antibody. Nuclear staining was performed with DAPI (SIGMA 09542).

Alkaline phosphatase staining

Alkaline phosphatase is an enzyme expressed by ESCs and is used as a marker of pluripotency. To evaluate the alkaline phosphatase expression, the cells were fixed in 10% Neutral Formalin Buffer for 15 min at 4°C, and washed three times with distilled water. These fixed cells were then incubated for 45 min at room temperature in 2ml of the staining solution prepared as follows: 0,005g Naphthol AS MX-PO4 (Sigma, N5000), 0,03g Red Violet LB salt (Sigma, F1625), 200 ml N, N-Dimethylformamide (DMF, Fischer Scientific, D1191), 25 ml of Tris-HCl (MW=157.6, pH 8.3, 0.2M), and 25 ml of distilled water The alkaline-phosphatase-positive cells showed a red color and were visible under phase-contrast microscopy.

RNA extraction and quantitative PCR detection of mRNA

RNA was extracted and purified using RNeasy kits (QIAGEN), according to the manufacturer instructions. Total RNA was treated with DNAse I (Qiagen) to prevent DNA contamination.

The cDNA was produced with SuperScript II Reverse Transcriptase kits (Life Technologies) starting from 1 μg mRNA. Real-time quantitative PCR reactions from 8,3 ng of cDNA was set up in triplicate using a LightCycle DNA SYBR Green I Master PCR machine (Roche). For oligos sequences see Supplementary Table 3. The primers for *Opa1* and *Drp1* and the primers for *Sdhb* and *Ndufs8* were previously described from Prieto and colleagues[9] and from Wu and colleagues[10]. For mitochondrial DNA analysis genomic DNA was extracted by using genomic DNA mini kit (Invitrogen) following manufacturer's instructions. mtDNA was measuread by real-time quantitative PCR reaction as the number of copies of the mitochondrial gene *tRNA-Tyr/mt-Co1* [11] normalized on the number of copies of *Gapdh*/2.

GENE	FORWARD	REVERSE
Gapdh	GTATGACTCCACTCACGGCAAA	TTCCATTCTCGGCCTTG
Nanog	AACCAAAGGATGAAGTGCAAG	TCCAAGTTGGGTTGGTCCA
Oct3/4	CGTGGAGACTTTGCAGCCTG	GCTTGGCAAACTGTTCTAGCTCCT
Peg3	CCCTCGACCATCTCATGC	TCTCGAGGCTCCACATCTCT
Rex1	AGGAAATAGGTAGAGCGCATCGCA	AGGCGATCCTGCTTTCTTCTGTGT
Opa1	ACAGCAAATTCAAGAGCACGA	TTGCGCTTCTGTTGGGCAT
Drp1	CAGGAATTGTTACGGTTCCTAA	CCTGAATTAACTTGTCCCGTGA
Ndufs8	GTTCATAGGGTCAGAGGTCAAG	TCCATTAAGATGTCCTGTGCG
Sdhb	ACCCCTTCTCTGTCTACCG	AATGCTCGCTTCTCCTTGTAG
Thy1	AACTCTTGGCACCATGAACC	TCAGGCTGGTCACCTTCTG
EpCAM	GCTGGCAACAAGTTGCTCTCTGAA	CGTTGCACTGGTTGGCTTTGAAGA
tRNA-Tyr/mt-Co1	CAGTCTAATGCTTACTCAGC	GGGCAGTTACGATAACATTG
genomic Gapdh	GAGCCAAAAGGGTCATCATC	AGTTGTCATGGATGACCTTGG

Supplementary Table 3. List of primers used for quantitative real-time PCR experiments.

Cell lysis and immunoblotting

Cells were harvested and washed twice with PBS. Cell lysis was performed on ice for 25 min, in RIPA buffer (150 mM NaCl, 1% Nonidet P40, 0.5% sodium deoxycholate, 0.1% sodium dodecyl sulphate, 50 mM Tris-HCl, pH 8.0) containing protease (SIGMA P8340) and phosphatase inhibitors (SIGMA P8340. Insoluble material was pelleted by centrifugation at 16,000× *g* for 3 min at 4 °C. Protein concentrations were determined using the Bradford assay (Bio-Rad 500-0006). The extract was mixed with 4×sample buffer (40% glycerol, 240 mM Tris/HCl, pH 6.8, 8% SDS, 0.04%

bromophenol blue, 5% b-mercaptoethanol), denatured at 99ºC for 10 minutes, separated by SDS-PAGE, and transferred to poly vinylidene difluoride membrane (BIO-RAD 162-0177). The membranes were blocked with 5% non-fat dry milk (SIGMA 70166) in TBS-Tween 20 (0,1%) (SIGMA P1379) for 60 min, incubated with primary antibody against rabbit PEG3 (ab99252), and mouse DRP1 (BD61112), OPA1 (BD612606), VDAC1 (ab14734), Total OXPHOS Antibody Cocktail (ab110413), SDHA (sc-390381), SDHB (sc-271548), Vinculin (V9131), and Tubulin (T0198) overnight at 4 °C, washed three times with TBS-T for 15 min, incubated with the peroxidase-conjugated secondary antibody (1:2000); Amersham Biosciences NA931V) in TBS-T with 5% non-fat dry milk for 60 min, and washed three times with TBS-T for 10 min. Immunoreactive proteins were detected using Pierce ECL Western Blotting Substrate (Thermo Scientific 32106). Densitometric analysis were carried out by using ImageJ software. The quantification reflects the relative amounts as a ratio of each protein band relative to their loading control.

Statistical Analysis.

Averages from three independent experiments were calculated for most of the shown experiments and Student's unpaired two-tailed t-test or one-way ANOVA were performed for statistical analysis: $p < 0.05$ defined statistical significance.

2. Supplementary References

1 Carey, B. W., Markoulaki, S., Beard, C., Hanna, J. & Jaenisch, R. Single-gene transgenic mouse strains for reprogramming adult somatic cells. *Nature methods* **7**, 56-59, doi:10.1038/nmeth.1410 (2010).

2 Conti, L. & Cattaneo, E. Controlling neural stem cell division within the adult subventricular zone: an APPealing job. *Trends Neurosci* **28**, 57-59, doi:10.1016/j.tins.2004.11.005 (2005).

3 Ying, Q. L., Nichols, J., Evans, E. P. & Smith, A. G. Changing potency by spontaneous fusion. *Nature* **416**, 545-548, doi:10.1038/nature729 (2002).

4 Schindelin, J. *et al.* Fiji: an open-source platform for biological-image analysis. *Nature methods* **9**, 676-682, doi:10.1038/nmeth.2019 (2012).

5 Valente, A. J., Maddalena, L. A., Robb, E. L., Moradi, F. & Stuart, J. A. A simple ImageJ macro tool for analyzing mitochondrial network morphology in mammalian cell culture. *Acta Histochem* **119**, 315-326, doi:10.1016/j.acthis.2017.03.001 (2017).

6 Ang, Y. S. *et al.* Wdr5 mediates self-renewal and reprogramming via the embryonic stem cell core transcriptional network. *Cell* **145**, 183-197, doi:10.1016/j.cell.2011.03.003 (2011).

7 Meerbrey, K. L. *et al.* The pINDUCER lentiviral toolkit for inducible RNA interference in vitro and in vivo. *Proceedings of the National Academy of Sciences of the United States of America* **108**, 3665-3670, doi:10.1073/pnas.1019736108 (2011).

8 Fuerer, C. & Nusse, R. Lentiviral vectors to probe and manipulate the Wnt signaling pathway. *PLoS One* **5**, e9370, doi:10.1371/journal.pone.0009370 (2010).

9 Prieto, J. *et al.* Early ERK1/2 activation promotes DRP1-dependent mitochondrial fission necessary for cell reprogramming. *Nature communications* **7**, 11124, doi:10.1038/ncomms11124 (2016).

10 Wu, Y. *et al.* Autophagy and mTORC1 regulate the stochastic phase of somatic cell reprogramming. *Nature cell biology* **17**, 715-725, doi:10.1038/ncb3172 (2015).

11 Facucho-Oliveira, J. M., Alderson, J., Spikings, E. C., Egginton, S. & St John, J. C. Mitochondrial DNA replication during differentiation of murine embryonic stem cells. *Journal of cell science* **120**, 4025-4034, doi:10.1242/jcs.016972 (2007).

3. Supplementary Figure legends

Supplementary Figure 1. Endogenous Peg3 levels anti-correlates with pluripotency state and its silencing enhances pluripotency marker expression (**A, C**) Quantitative real-time PCR analysis of endogenous *Peg3* expression levels during EB (**A**) and NSC (**C**) differentiation (n=3 independent experiments). (**B, D**) Representative western-blots (out of n=2 independent experiments) showing endogenous PEG3 protein levels in ESC-to-EB (**B**) and ESC-to-NSC (**D**) differentiation. (**E**) Quantitative real-time PCR analysis showing *Peg3* silencing efficiency (left) and pluripotency gene (*Nanog*, *Oct4)* expression levels after shPeg3, shOct3/4 and shCtrl transfection (n=3 independent experiments). (**F, G**) FACS quantification of GFP^{+} and GFP^{-} percentage of REX1-dGFP ESCs in serum-Lif (Serum) *versus* 2i-Lif medium (2i-Lif) (**F**) and during NSC differentiation (**G**) after shCtrl and shPeg3 transduction (n=3 independent experiments). (**H**, **I**) Quantitative real-time PCR (n=3 technical replicates) (**H**) and representative western-blot analysis (n=1) showing PEG3 silencing in ESCs at day 0 and 3 of NSC differentiation (**I**). Data are represented as fold change (2^-ΔΔCt) (**A**, **C**, **E**) and means (F, G) of n=3 independent experiments ± SE. Asterisks indicate statistical significance calculated by unpaired two-tailed t test analysis (**E, F, G**) and by one-way ANOVA (**A**, **C**) (*p < 0.05; ** p < 0.01; *** p < 0.001). For western blot analysis β-Tubulin was used as loading control and densitometric analysis were carried out by using ImageJ software. The quantification reflects the relative amounts as a ratio of each protein band relative to their loading control.

Supplementary Figure 2. Peg3 inducible silencing enhanced the efficiency of both MEF and NSC reprogramming. (**A, B**) Representative FACS plot analysis showing E-cadherin expression at day 0 (left) and 12 (right) of OKS reprogramming (**A**) and its quantification (n=3 technical replicates) (**B**). (**C**) Representative immunofluorescence image showing NANOG expression in NSC derived iPSCs. (**D**) Representative scheme of the pINDUCER-shPeg3 (pIND-shPeg3) lentiviral vector. (**E**) Representative bright field and fluorescence microscopy images of ESCs infected with pIND-shPeg3, selected with puromycin and treated with DOX for 48h. (**F**) FACS analysis showing RFP signal of ESCs after puromycin selection and 48h of DOX treatment. (**G**) Quantitative real-time PCR analysis of the pIND-shPeg3 silencing and of the pluripotency gene expression after DOX treatment. Data are represented as fold change and means of n=3 technical replicates ± SD. (**H**) Experimental scheme of wild type (WT) MEF and NSC to iPSC reprogramming. MEFs and NSCs were infected with pIND-shPeg3 and with OKSM and OKM respectively and re-plated in equal number. The following day the media was switched to ESCs media ± DOX. The number of $NANOG^+$ iPSC clones was counted at day 16 of reprogramming. (**I, J**) Representative fluorescence microscopy images of $NANOG^+$ iPSC clones obtained from WT MEFs (**I**) and NSCs (**J**). (**K, L**) Number of NANOG+ clones counted obtained from WT MEFs (**K**) and NSCs (**L**) at day 16 of reprogramming ± DOX (represented as fold change of n=3 independent experiments). (**M, N)** Quantitative real-time PCR analysis showing *Peg3* silencing efficiency of the pIND-shPeg3 construct (-DOX *versus* +DOX) at day 5 of MEF (**M**) and NSC (**N**) reprogramming (n=3 independent experiments). (**K, L, M, N**) Data are represented as fold change (2^-ΔΔCt) and means of n=3 independent experiments ± SE. Asterisks indicate statistical significance calculated by unpaired two tailed t test analysis (*p <

0.05; ** $p < 0.01$; (*$p < 0.05$; ** $p < 0.01$; *** $p < 0.001$). Nuclei were stained with DAPI. Scale bar is 200 µm (**E**), 100 µm (**I**) and 50 µm (**C**, **J**).

Supplementary Figure 3. Peg3 transient overexpression does not cause apoptosis and causes minor ESC self-renewal impairment. (**A**) Experimental scheme showing EF1α-EV-SV40-mCherry and EF1α-Peg3-SV40-mCherry transfection. (**B**) FACS-analysis plots showing the efficiency of transfection according to the mCHERRY signal. ESCs were transfected with either EF1α-EV-SV40-mCHERRY or EF1α-Peg3-SV40-mCHERRY and the non transfected (NT) ESCs were used as a control. (**C**) Representative western-blot (out of n=2 independent experiments) showing PEG3 protein levels 48 hours after EF1α-EV-SV40-mCHERRY or EF1α-Peg3-SV40-mCHERRY ESC transfection. (**D)** Representative AP^{+}-CFC assay after either EV or PEG3 transfection. 24h after transfection 10^{2} cells/cm^{2} for each condition were plated and cultured for additional 5 days. (**E**) AP^{+} colonies counted at day 5 of AP^{+}-CFC assay. Data are represented as means of n=3 independent experiments ± SE (**F, G**) Representative FACS plot showing AnnexinV staining of ESCs transfected either with EV (**F**) or PEG3 (**G**) analyzed at 24h and 48h. The non stained (NS) control cells were included in the analysis. β-Tubulin was used as loading control and densitometric analysis were carried out by using ImageJ software. The quantification reflects the relative amounts as a ratio of each protein band relative to their loading control.

Supplementary Figure 4. Peg3 silencing correlates with mitochondria activity downregulation. (**A**) Representative FACS profile and quantification (n=3 independent experiments) of tetradmethylrhodamine ethyl ester (TMRE) intensity in ESCs transduced with either shCtrl or shPeg3 and selected with hygromycin. (**B**) Representative FACS plot and quantification (n=3 independent experiments) of TMRE intensity in ESCs treated for 16h with either ethanol (EtOH) or with the uncoupler carbonyl cyanide-m-chlorophenylhydrazone (CCCP). (**C**) Representative bright field images of shCtrl (left) and shPeg3 (right) transduced EBs at day 5 of the differentiation protocol. (**D**, **E**) Quantitative real-time PCR analysis showing expression levels of differentiation markers (**D**) and of mitochondrial gene expression level (**E**). Data are represented as means of n=3 technical replicates ± SD. (**F, G**) Box plot representing median mitochondrial branch length quantification (corresponding to the middlemost value of all branches lengths) in shCtrl and shPeg3 infected MEFs and day 0 (**F**) and 5 (**G**) of reprogramming. (**H, I**) Representative FACS profile showing MitoTracker Green (MTG) intensity in MEFs transduced with shCtrl and shPeg3 measured at day 0 (**H**) and day 5 (**I**) of reprogramming. (**J, K**) Representative FACS profile showing TMRE intensity in MEFs transduced with shCtrl and shPeg3 at day 0 (**J**) and day 5 (**K**) of reprogramming. (**L**) Quantification of TMRE signaling from day 0 to day 5 of reprogramming in MEFs transduced either with shCtrl or shPeg3. (**A**, **B**, **L**) Data are represented as means of n=3 independent experiments ± SE and asterisks indicate statistical significance calculated by unpaired two tailed t test analysis (*p < 0.05; ** p < 0.01; *** p < 0.001).

Supplementary Figure 5. Peg3 overexpression correlates with higher mitochondria activity. (**A**) Representative western-blot (out of 2 independent experiments) showing the level of PEG3 protein in EV and PEG3 overexpressing ESCs. (**B**) Quantification of MTG intensity in ESC transfected with either EV or PEG3 (n=3 independent experiments). (**C**) Representative western-blot (out of 2 independent experiments) showing the level of mitochondrial protein VDAC1 in EV and PEG3 overexpressing ESCs. (**D, E**) Representative western-blot showing the levels of OXPHOS protein complexes (out of 2 independent experiments) (**D**), and SDHA and SDHB proteins (n=1) (**E**) in EV and PEG3 overexpressing ESCs. (**F**) Quantitative real-time PCR analysis showing mitochondrial DNA quantification in ESCs calculated over *Gapdh* genomic DNA region (n=3 independent experiments). (**G**) Representative western-blot (out of 2 independent experiments) showing PEG3 overexpression levels in HEK293T cells. (**H**) Quantification of MTG intensity in ESC transfected with either EV or PEG3 (n=3 independent experiments). For western blot analysis β-Tubulin was used as loading control and densitometric analysis were carried out by using ImageJ software. The quantification reflects the relative amounts as a ratio of each protein band relative to their loading control. (**B**, **F, H**) Data are represented as means of n=3 independent experiments ± SE and asterisks indicate statistical significance calculated by unpaired two-tailed t test analysis (*p < 0.05)

Supplementary Figures

Supplementary Figure 1

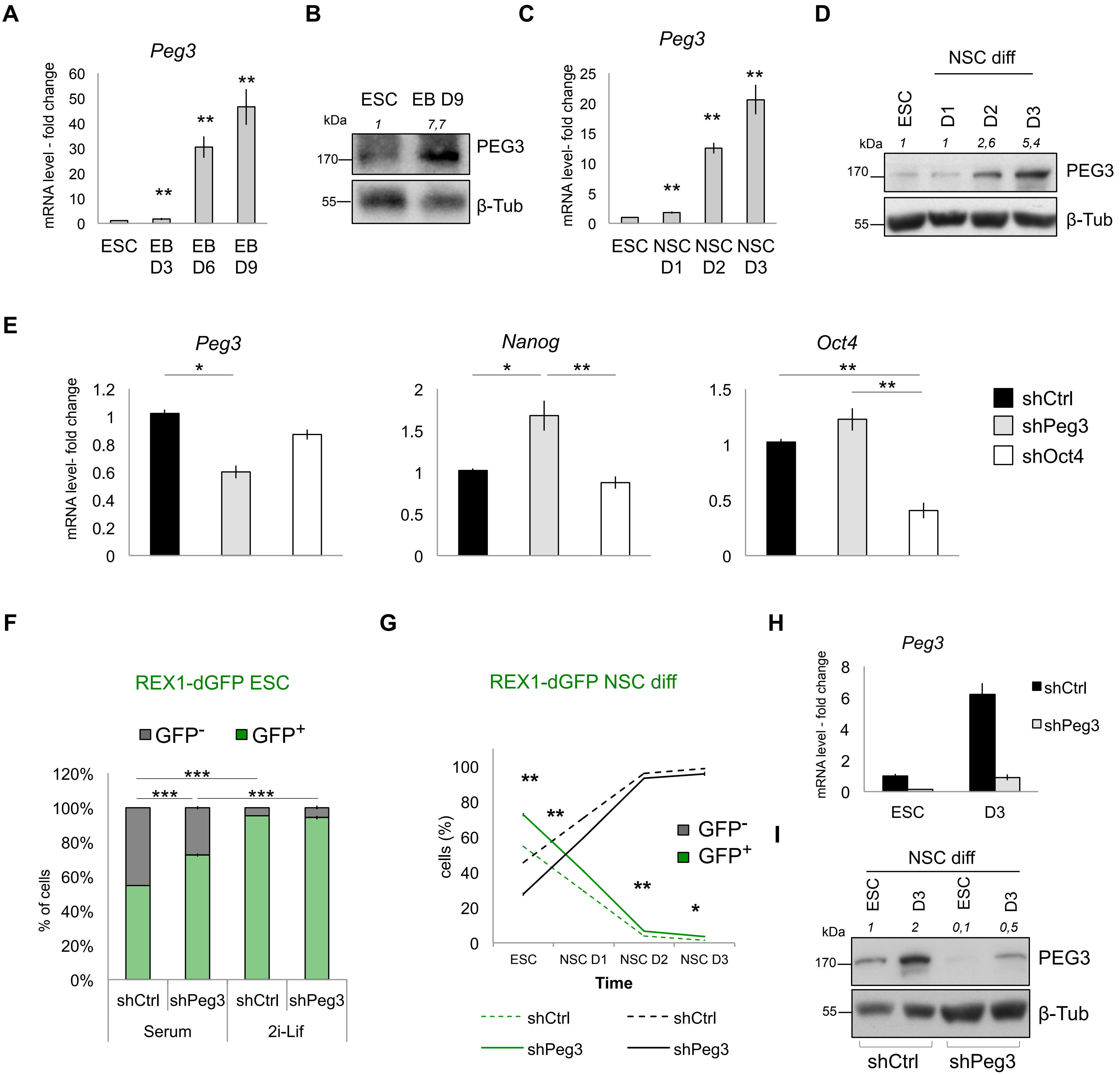

Supplementary Figure 2

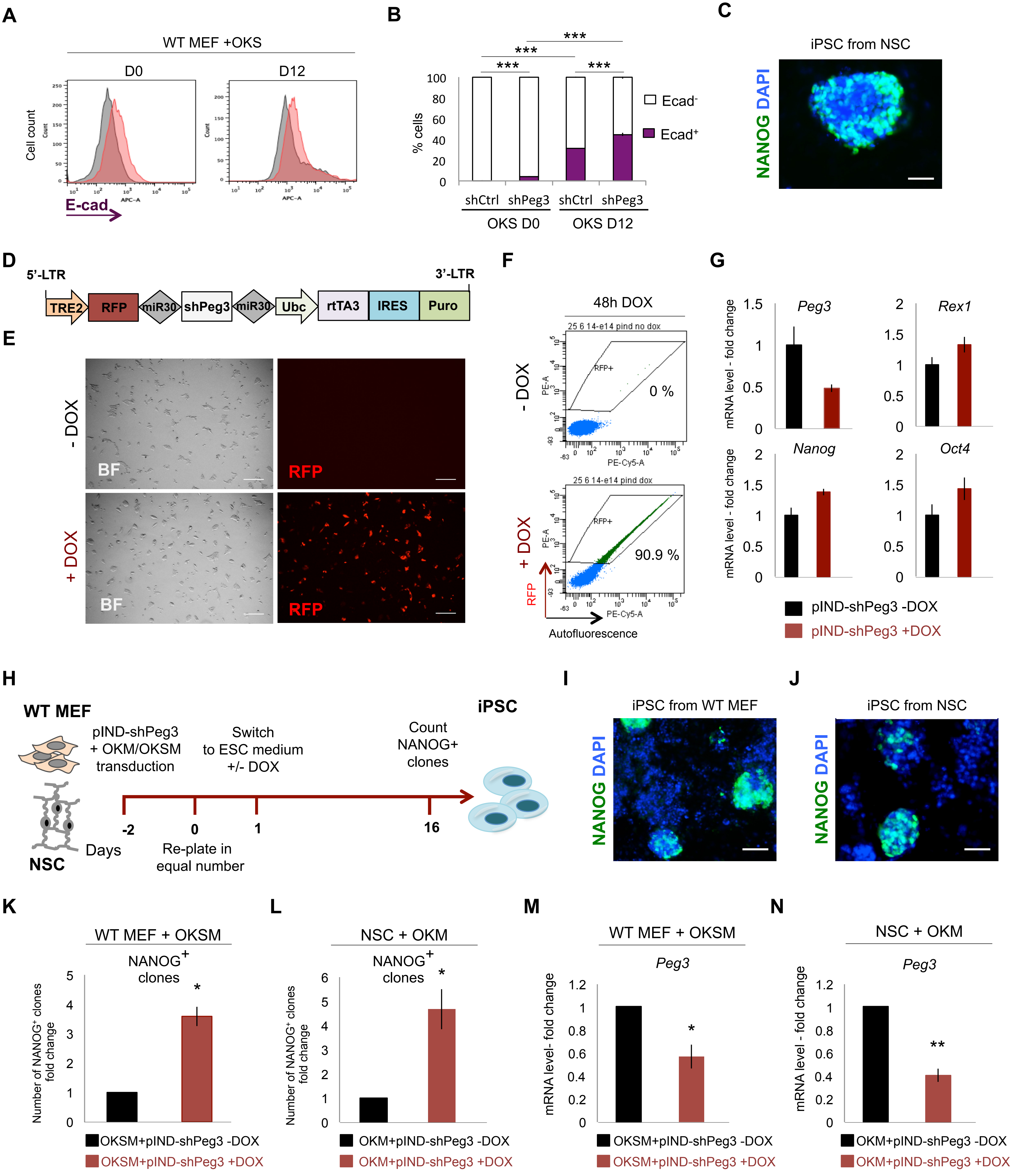

Supplementary Figure 3

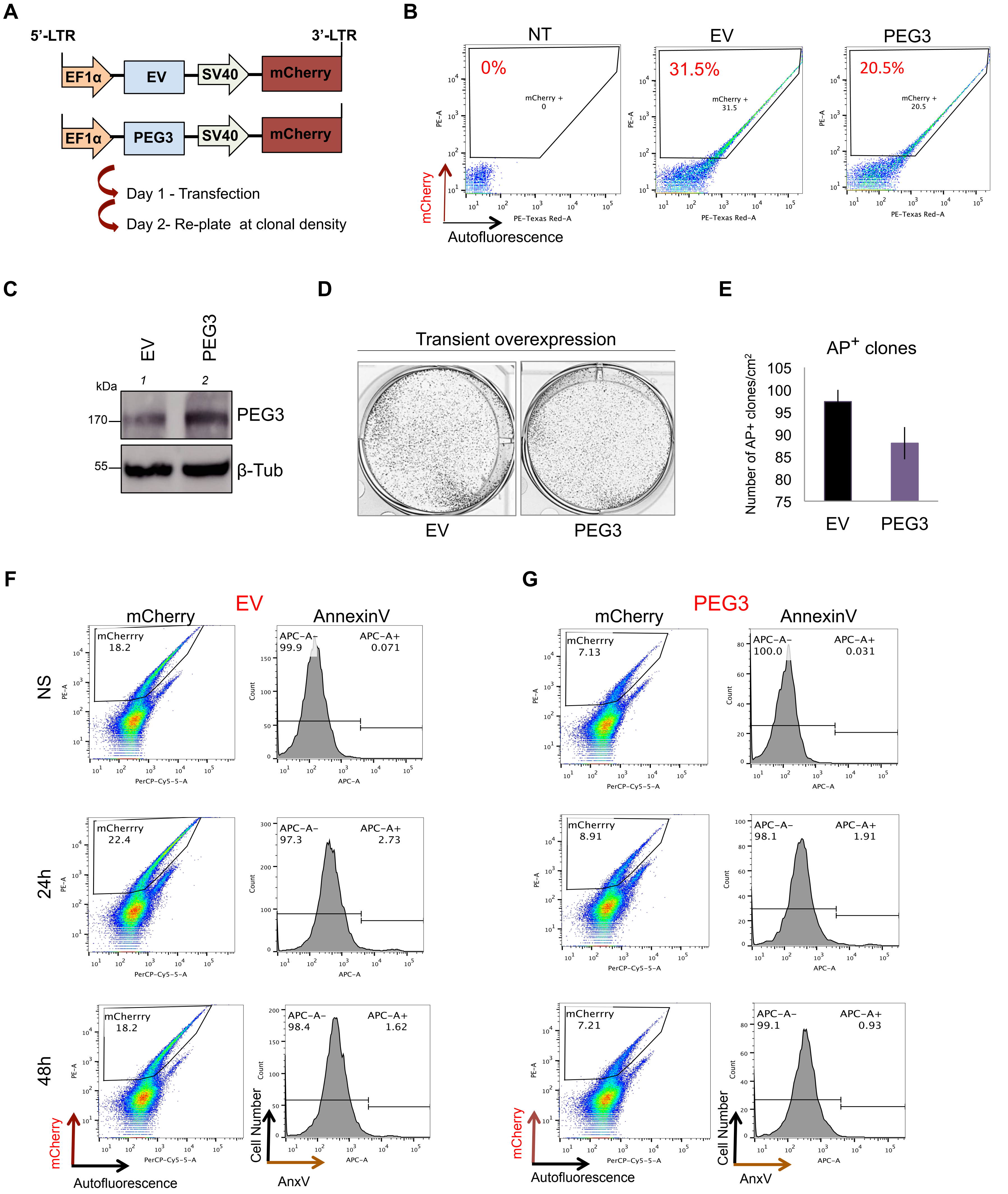

Supplementary Figure 4

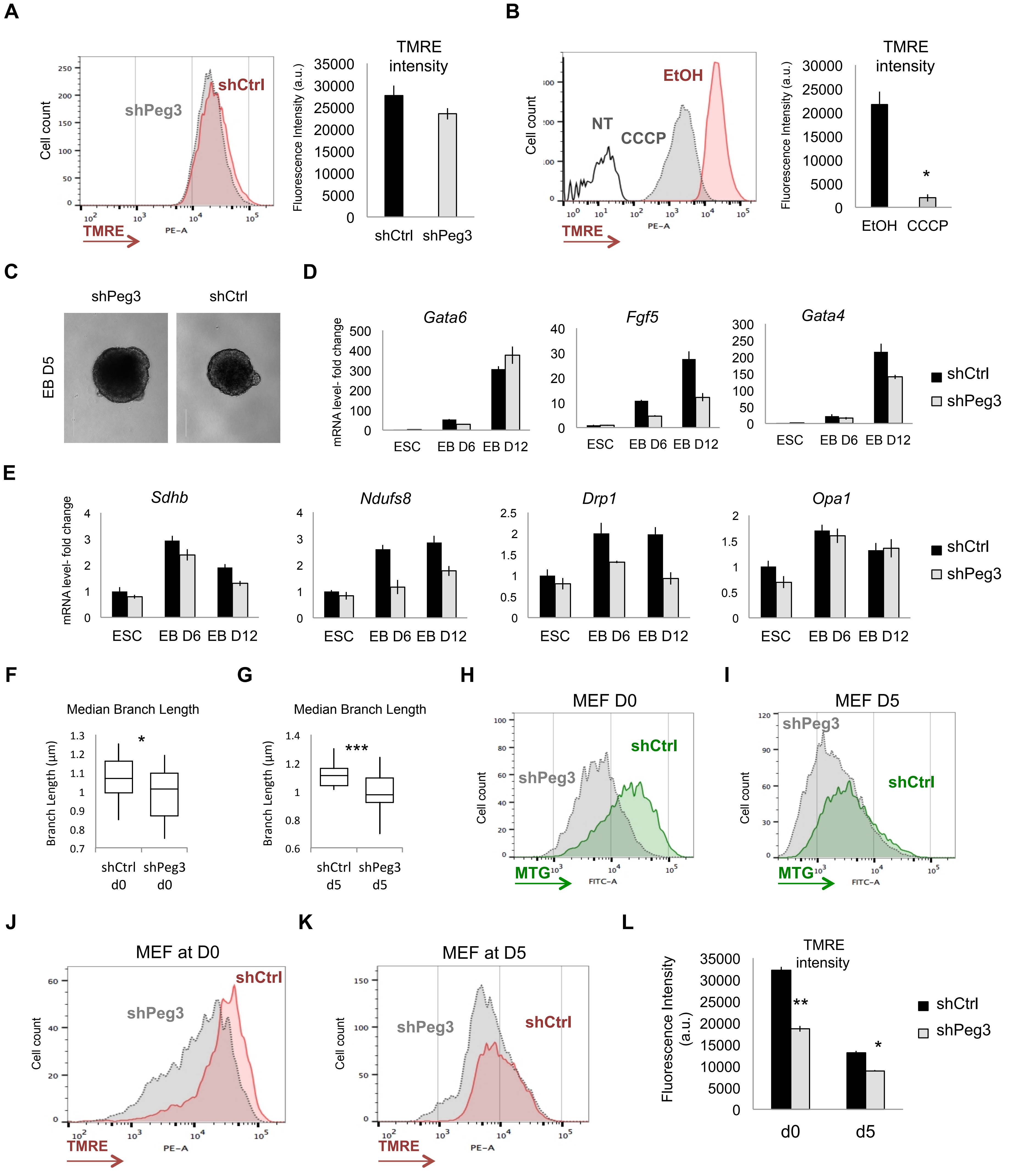

Supplementary Figure 5

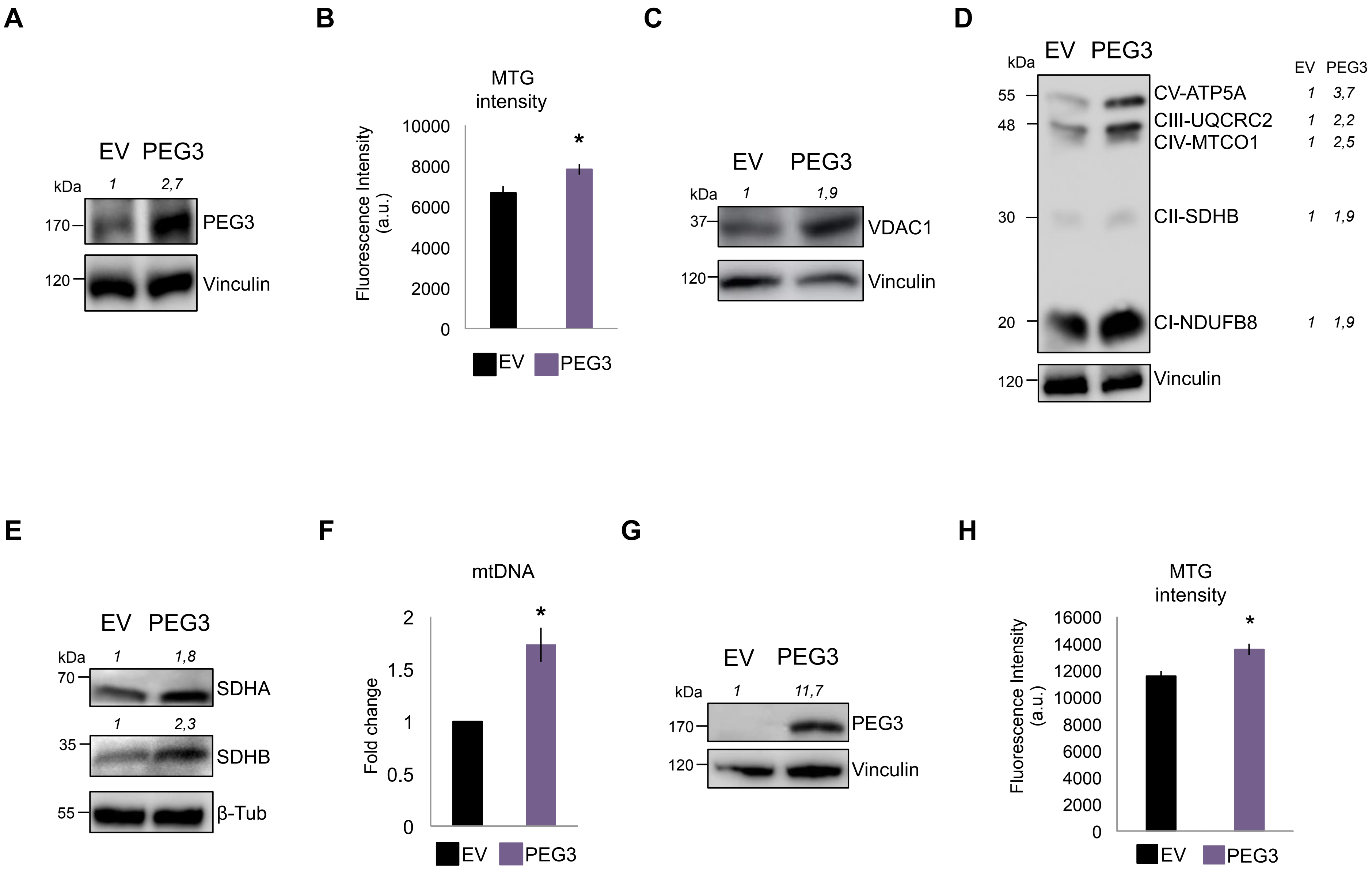

Supplement: Supplementary file 1 — Supplementary information and figures [file 41598_2017_10016_MOESM1_ESM.pdf]
